# Unveiling inflammatory and prehypertrophic cell populations as key contributors to knee cartilage degeneration in osteoarthritis using multi-omics data integration

**DOI:** 10.1136/ard-2023-224420

**Published:** 2024-02-07

**Authors:** Yue Fan, Xuzhao Bian, Xiaogao Meng, Lei Li, Laiyi Fu, Yanan Zhang, Long Wang, Yan Zhang, Dalong Gao, Xiong Guo, Mikko Juhani Lammi, Guangdun Peng, Shiquan Sun

**Affiliations:** 1 Center for Single-Cell Omics and Health, School of Public Health, Xi'an Jiaotong University Health Science Center, Xi'an, Shaanxi, China; 2 Collaborative Innovation Center of Endemic Diseases and Health Promotion in Silk Road Region, Shaanxi Province; Key Laboratory of Trace Elements and Endemic Diseases, Xi'an Jiaotong University, Xi'an, Shaanxi, China; 3 Key Laboratory of Environment and Genes Related to Diseases, Ministry of Education; Key Laboratory for Disease Prevention and Control and Health Promotion of Shaanxi Province, Xi'an Jiaotong University, Xi'an, Shaanxi, China; 4 Guangzhou Institutes of Biomedicine and Health, Chinese Academy of Sciences, Guangzhou, China; 5 Division of Life Sciences and Medicine, University of Science and Technology of China, Hefei, Anhui, China; 6 Center for Cell Lineage and Development, CAS Key Laboratory of Regenerative Biology, Guangdong Provincial Key Laboratory of Stem Cell and Regenerative Medicine, GIBH-HKU Guangdong-Hong Kong Stem Cell and Regenerative Medicine Research Centre, China-New Zealand Joint Laboratory on Biomedicine and Health, Guangzhou Institutes of Biomedicine and Health, Chinese Academy of Sciences, Guangzhou, Guangdong, China; 7 School of Automation Science and Engineering, Xi'an Jiaotong University, Xi'an, Shaanxi, China; 8 Center for Evidence-Based Medicine and Clinical Research, Taihe Hospital, Hubei University of Medicine, Shiyan, Hubei, China; 9 Department of Orthopaedics, Honghui Hospital, Xi'an, Shaanxi, China; 10 Department of Orthopaedics, The Central Hospital of Xianyang, Xianyang, China; 11 Department of Integrative Medical Biology, University of Umeå, Umeå, Sweden; 12 University of Chinese Academy of Sciences, Beijing, China

**Keywords:** inflammation, chondrocytes, osteoarthritis, knee

## Abstract

**Objectives:**

Single-cell and spatial transcriptomics analysis of human knee articular cartilage tissue to present a comprehensive transcriptome landscape and osteoarthritis (OA)-critical cell populations.

**Methods:**

Single-cell RNA sequencing and spatially resolved transcriptomic technology have been applied to characterise the cellular heterogeneity of human knee articular cartilage which were collected from 8 OA donors, and 3 non-OA control donors, and a total of 19 samples. The novel chondrocyte population and marker genes of interest were validated by immunohistochemistry staining, quantitative real-time PCR, etc. The OA-critical cell populations were validated through integrative analyses of publicly available bulk RNA sequencing data and large-scale genome-wide association studies.

**Results:**

We identified 33 cell population-specific marker genes that define 11 chondrocyte populations, including 9 known populations and 2 new populations, that is, pre-inflammatory chondrocyte population (preInfC) and inflammatory chondrocyte population (InfC). The novel findings that make this an important addition to the literature include: (1) the novel InfC activates the mediator MIF-CD74; (2) the prehypertrophic chondrocyte (preHTC) and hypertrophic chondrocyte (HTC) are potentially OA-critical cell populations; (3) most OA-associated differentially expressed genes reside in the articular surface and superficial zone; (4) the prefibrocartilage chondrocyte (preFC) population is a major contributor to the stratification of patients with OA, resulting in both an inflammatory-related subtype and a non-inflammatory-related subtype.

**Conclusions:**

Our results highlight InfC, preHTC, preFC and HTC as potential cell populations to target for therapy. Also, we conclude that profiling of those cell populations in patients might be used to stratify patient populations for defining cohorts for clinical trials and precision medicine.

**WHAT IS ALREADY KNOWN ON THIS TOPIC**

- Recent single-cell omics studies have comprehensively presented the cellular heterogeneity of chondrocytes that exhibit 11 putative cell populations, rather than the long-held view that cartilage consists of only a single dominant cell type, chondrocytes.

**WHAT THIS STUDY ADDS**

- We present a transcriptome landscape of human knee articular cartilage using single-cell RNA sequencing (scRNA-seq) and spatially resolved transcriptomic data.
- We carefully curate a cell marker gene list for osteoarthritis (OA) or non-OA human cartilage that is capable of annotating all existing scRNA-seq data.
- We identify a novel inflammatory cell population (InfC) that potentially activates the ligand-receptor pair MIF-CD74 in knee cartilage degeneration in OA.
- We highlight that prehypertrophic chondrocyte (preHTC) is transcriptionally associated with OA, prefibrocartilage chondrocyte (preFC) is potentially OA-critical cell populations that contribute to the subtyping of patients with OA and HTC is genetically related to the genome-wide association studies variants in large-scale UK Biobank studies.

**HOW THIS STUDY MIGHT AFFECT RESEARCH, PRACTICE OR POLICY**

- Our research has offered valuable insights into the pathogenesis of OA, highlighting four key cell populations—InfC, preHTC, preFC and HTC—that could be targeted for therapeutic intervention.

## Introduction

Osteoarthritis (OA) of the knee is known to be an age-related, multifactorial and high-prevalence heterogeneous joint disorder.^1 2^ It is typically characterised by articular cartilage degeneration,^3^ cartilage calcification or mineralisation^4^ and subchondral bone sclerosis,^5^ as well as inflammatory responses.^6–9^ One of the hurdles in curbing the progression of OA is the considerable heterogeneity among subtypes of patients or cell populations of chondrocytes, making it difficult to find a single target (eg, a specific cell population or biomarker) for disease interventions.^10 11^ Over the past decade, traditional multi-omics studies have been primarily conducted to understand the molecular processes that are potentially involved in disease progression at the population level.^12^ For example, genomic data were used to identify OA-associated genetic variants,^13 14^ such as rs375575359 and rs11335718; transcriptomic data were used to detect key molecular differences or characterise the subtype of patients with OA,^15^ such as *MMP13*, *MMP2* and *CYTL1*; epigenomic data were used to reflect how epigenetic modifications that regulate gene expression,^16^ and proteomic data were used to determine what changes in genomics, transcriptomics or epigenomics,^17 18^ such as CRTAC1 protein in plasma was considered as a promising candidate biomarker for OA. However, conventional multi-omics sequencing technologies are only capable of measuring the average expression level of hundreds of thousands of cells together,^19 20^ largely ignoring the cellular heterogeneity of chondrocytes in articular cartilage tissues.

With the emergence of single-cell sequencing technologies^21 22^ and spatially resolved technologies,^23^ unbiased and high-throughput profiling of tissue architecture at single-cell and spatial resolution is now feasible. This presents an unprecedented opportunity to uncover the cellular or spatial heterogeneity of cartilage tissue and delineate the differentiation trajectories of chondrocytes; therefore, greatly facilitating the identification of novel or rare chondrocyte populations for maintaining cartilage homeostasis and preventing the development and progression of OA. Recently, several single-cell omics studies have been conducted to unravel the cellular heterogeneity of cartilage tissues,^10 24 25^ revealing that chondrocytes in cartilage can exhibit diverse compositions, rather than the long-held view that cartilage consists of only a single dominant cell type. Of note, the role of chondrocytes depends on the different types of cell populations, and on the spatial nature of cartilage architecture,^26 27^ orchestrated by articular surface (AS), superficial zone (SZ), middle zone (MZ) and deep zone (DZ). Therefore, a comprehensive characterisation of chondrocyte composition and the spatial landscape of cartilage tissue in a high-resolution, untargeted and unbiased manner is an important step towards the understanding of the molecular mechanisms of chondrocyte differentiation, proliferation,^28^ senescence,^29^ autophagy^30^ and apoptosis^31^ in patients with OA. In light of this, a comprehensive transcriptional landscape of chondrocytes is urgently needed to systematically investigate cellular heterogeneity and spatial heterogeneity.

Meanwhile, numerous large-scale traditional multi-omics studies involving genome-wide association studies (GWAS) and bulk RNA sequencing (RNA-seq) studies have been performed, identifying >100 variants^14 32–34^ and a plethora of OA-associated differentially expressed (DE) genes^11^ that potentially relate to the pathogenic mechanisms and drug targets of OA. However, due to cellular heterogeneity and interpatient variances, it remains unknown which molecular subtypes of chondrocyte populations represent the reproducible stratification of patients with OA, and which chondrocyte populations may essentially contribute to the development and progression of OA.

Here, we collected 19 cartilage samples from 8 patients with OA and 3 normal controls and profiled a total of 135 896 chondrocytes by the droplet-based single-cell RNA sequencing (scRNA-seq) protocol (ie, 10x Genomics^35^) to investigate the cellular heterogeneity of human knee articular cartilage tissue. Additionally, 124 samples of 9 cartilage samples from 4 patients with OA and 1 normal control are subjected to spatial transcriptomic analysis by laser microdissection-based RNA sequencing (Geo-seq^36^) to investigate the spatial heterogeneity of human knee articular cartilage tissue. We performed an integrative analysis of scRNA-seq data and Geo-seq data to define the spatial landscape of chondrocytes, to identify zone-specific genes, to detect zone-specific DE genes and to enrich the functional pathways associated with markers and DE genes. Next, we performed an integrative analysis of scRNA-seq data and two bulk RNA-seq data to stratify patients with OA into two distinct subtypes. Finally, we combined the scRNA-seq data with two large-scale GWAS to infer the OA-critical chondrocyte populations that related to genetic variants and chondrocyte populations that confer the risk of OA. Overall, our findings revealed the prominent role of critical cell populations and tissue architectures in the pathophysiology of OA, which will greatly expand our understanding of the populations and cellular heterogeneity of cartilage tissue from patients with OA and non-OA controls, and stratify the subtypes of patients with OA for the design of clinical trials.

## Results

### Single-cell transcriptional landscape of human knee cartilage in patients with OA and non-OA controls

**Figure 1:**
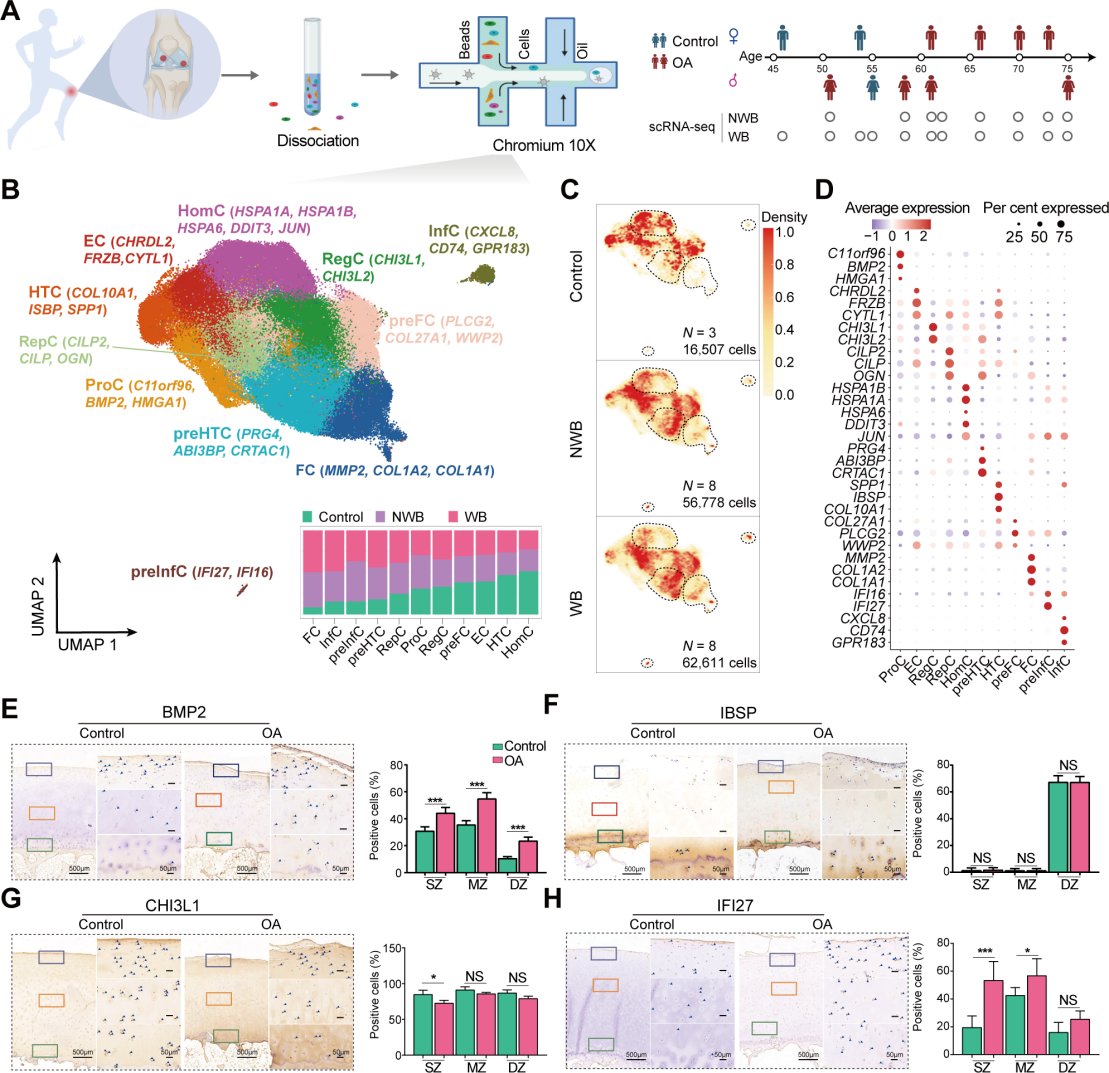
The single cell landscape of human knee cartilage in WB, NWB and non-OA controls. (A) Schematic of workflows for transcriptomic profiling of human articular cartilage using scRNA-seq. The scRNA-seq data (n=11, 5 females and 6 males) were assayed by 10x Genomics protocols, including 8 paired WB and NWB samples, and 3 non-OA controls. (B) The top panel shows the visualisation of chondrocyte populations using UMAP, where a total of 135 896 chondrocytes were analysed by the integrative analysis of 8 paired WB and NWB samples and 3 non-OA controls. There is a total of 11 populations, that is, RegC (regulator chondrocytes, green), ProC (proliferation chondrocytes, yellow), HomC (homeostasis chondrocytes, purple), preHTC (azure), EC (effector chondrocytes, red), FC (fibrocartilage chondrocytes, blue), HTC (orange), RepC (reparative chondrocytes, aqua), preFC (pink), preInfC (crimson) and InfC (brown). Each cell population was annotated by a given marker gene list explored in this study. The preInfC and InfC are new chondrocyte populations. The bar plot at the bottom panel shows the corresponding cell compositions among WB, NWB and non-OA control samples. The cell composition is similar between WB and NWB, while largely different with non-OA control samples. Proportions were scaled by the total number of cells assayed. (C) The cell density plot shows the visualisation of the average cell density within the non-OA control (upper panel), NWB (middle panel) and WB (bottom panel). Regions with higher density are represented by darker colours. The preHTC, InfC, preInfC and FC are strongly enriched in NWB and WB. (D) The dot plot shows the mean expressions of the marker genes for 11 major cell populations. The colour represents the average scaled gene expression level (z-score) and the dot size represents the percentage of cells that the marker gene detected in each cell population. (E) The marker gene of ProC, BMP2, is validated by IHC staining. (F) The marker gene of HTC, IBSP, is validated by IHC. (G) The marker gene of RegC, CHI3L1, is validated by IHC. (H) The marker gene of preInfC, IFI27, is validated by IHC. The quantification of positive cells from different zones (ie, SZ, MZ and DZ) is displayed by bar plots (replicates n=6), where the AS and SZ were hardly distinguished during IHC staining, therefore, the AS and SZ in Geo-seq results were merged together to only present as SZ in IHC staining. The scale bar: left, 500 μm; right, 50 μm. AS, articular surface; DZ, deep zone; Geo-seq, laser microdissection-based RNA sequencing; IHC, immunohistochemistry; InfC, inflammatory chondrocyte; MZ, middle zone; NS, no significant; NWB, non-weight-bearing; OA, osteoarthritis; preFC, prefibrocartilage chondrocyte; preHTC, prehypertrophic chondrocyte; preInfC, pre-inflammatory chondrocyte; scRNA-seq, single-cell RNA sequencing; SZ, superficial zone; UMAP, uniform manifold approximation and projection; WB, weight-bearing. *p<0.05, ***p<0.001.

To comprehensively present the cellular landscape of knee cartilage tissue, we measured the single-cell transcriptomic profiles of chondrocytes from a total of 19 cartilage samples (online supplemental table S1; n=135 896 chondrocytes after the stringent quality control, online supplemental table S2), which were collected from 8 patients with OA (n=119 389 chondrocytes) and 3 non-OA controls (n=16 507 chondrocytes). In particular, the OA samples were split into weight-bearing (WB) cartilage (n=62 611 chondrocytes) from the medial condyle of the femur and non-weight-bearing (NWB) cartilage (n=56 778 chondrocytes) from the intact lateral condyle of the femur to serve as internal controls (figure 1A). The transcriptomic profiling of chondrocytes was profiled through 10x Genomics^35^ (see ‘Materials and methods’ section).

10.1136/ard-2023-224420.supp2 Supplementary data

We performed an integrative analysis of multiple scRNA-seq datasets to investigate the cellular heterogeneity of chondrocytes from multiple samples (online supplemental figure S1A) and finally classified the chondrocytes into a total of 11 putative cell populations (figure 1B). Specifically, we observed four cell populations (ie, FC, InfC, preInfC and preHTC) were dominated in the patients with OA (ie, WB and NWB); while one cell population (ie, HomC) was dominated in non-OA controls (figure 1C). Notably, we found that two novel chondrocyte populations, pre-inflammatory chondrocytes (preInfC, 533 cells; *IFI16* and *IFI27*) and inflammatory chondrocytes (InfC, 1499 cells; *CD74*, *CXCL8* and *GPR183*) were strongly enriched in both WB and NWB samples (WB>NWB; figure 1C), indicating these two populations may potentially contribute to the pathogenesis of OA.

10.1136/ard-2023-224420.supp1 Supplementary data

Next, we performed a trajectory inference analysis to examine the differentiation relationship of these populations (see ‘Materials and methods’ section). We found that all chondrocytes from patients with OA and non-OA controls were distributed along a single lineage trajectory (online supplemental figure S1B). Consistent with the previous studies,^10^ ProCs were initially located at the start of the trajectory, followed by HTCs, ECs and HomCs. Whereas FCs, preInfCs and InfCs were mainly distributed in the termination of the trajectory, indicating that chondrocytes exhibited fibrosis and inflammation at the differentiated destination, which is one of the hallmarks of OA.^37 38^

### Cell population-specific marker genes for annotating chondrocytes of cartilage tissue

The chondrocytes of knee cartilage generally demonstrated a high correlation in intrapopulations (online supplemental figure S2A) but a low correlation in intersamples (online supplemental figure S2B), resulting in a great challenge to identify cell population-specific marker genes to annotate the putative chondrocyte populations. As far as we know, apart from the canonical markers, for example, *COL10A1* for HTC; *COL1A1* and *COL1A2* for FC, there have not been any marker genes available that can reliably distinguish putative chondrocyte populations. With the large-scale and multisource scRNA-seq data, we finally identified 33 marker genes from the top 50 population-specific marker genes (online supplemental table S3), which were applicable for annotating cell populations under different cartilage statuses (online supplemental figure S1C).

Specifically, we found that the gene *C11orf96*, *BMP2* and *HMGA1* exhibited distinct expression levels for ProC (figure 1D). ProC was enriched in the expression of genes related to chondrocyte differentiation, wound healing and cytoplasmic translation pathways (online supplemental figure S2C; online supplemental table S4) and marked by transcription factors, FOSL1 and RELB (online supplemental figure S2D). To validate the presence of ProCs in cartilage tissue, we performed BMP2 IHC staining on patients with OA and non-OA controls. *BMP2* as a member of the transforming growth factor-β (pro-inflammatory cytokines) superfamily and an anabolic factor, was positively observed throughout all zones of knee articular cartilage (figure 1E), implying these chondrocytes were effective in promoting cartilage repair and regeneration.^39^

Although *COL10A1* is a canonical marker for HTC, we also expanded another two markers *IBSP* and *SPP1* as candidates for HTC and there two markers have been demonstrated to be highly expressed in the terminal hypertrophic chondrocytes.^40^ HTC was enriched in the expression of genes related to extracellular matrix (ECM) organisation and ossification (online supplemental figure S2C; online supplemental table S4). The IHC staining confirmed that IBSP was highly expressed in the DZ of patients with OA and non-OA controls (figure 1F). Meanwhile, we identified both *CHI3L1* and *CHI3L2* as markers for RegC that related to some catabolic process such as amino sugar catabolic process (online supplemental figure S2C; online supplemental table S4). To validate the expression levels of CHI3L1, we also performed IHC staining on patients with OA and non-OA controls (figure 1G).

In addition, we defined new markers for preFC, that is, *COL27A1, WWP2* and *PLCG2*. Where *COL27A1* (Chr9:116,909,146 T/A, p=3.4e-13) was genetically associated with knee pain in large-scale GWAS^41^ and *WWP2* is essential for maintaining cartilage homeostasis and protecting cartilage against OA.^42^ Therefore, preFC may potentially be a critical cell population in alleviating cartilage lesions.

Notably, the other two novel cell populations, that is, preInfC and InfC were uniquely identified in the patients with OA. Specifically, preInfC highly expressed marker gene *IFI27*, which was related to apoptosis^43^ and angiogenesis.^44 45^ Subsequently, we performed IFI27 IHC staining on patients with OA and non-OA controls, demonstrating that IFI27 is highly expressed in the SZ of patients with OA (p=4.0e-4, figure 1H).

In summary, we identified 33 representative marker genes for annotating putative chondrocyte populations (table 1). To validate the efficacy of these candidate markers in annotating cell populations, we applied them to another two publicly available scRNA-seq datasets.^10 25^ The results demonstrated the effectiveness of the marker gene set in accurately annotating cell populations across distinct sequencing technologies, including Smart-seq2^10^ (online supplemental figure S3A) and 10x Genomics^25^ (online supplemental figure S3B). Notably, the previously defined marker genes from studies^10 25^ did not exhibit consistent and accurate annotation of scRNA-seq data in this research (online supplemental figures S3C and S3D).

**Table 1:** The 33 marker genes are used for annotating 11 distinct chondrocyte populations

Population	Marker gene	Function
ProC	***C11orf96***, ***BMP2*** and ***HMGA1***	Chondrocyte differentiation, wound healing, cytoplasmic translation
EC	*CHRDL2,* 10 ***FRZB*** and *CYTL1* 25	Cartilage development
RegC	*CHI3L1* 25 and *CHI3L2* 25	Response to metal ion, amino sugar catabolic process
RepC	***CILP2*** *, CILP* 94 and ***OGN***	Cartilage development and condensation, chondrocyte differentiation
HomC	***HSPA1B***, ***HSPA1A***, ***HSPA6*** *, DDIT3* 10 and *JUN* 10	Response to unfolded protein, response to heat
preHTC	***PRG4***, ***ABI3BP*** and ***CRTAC1***	ECM organisation, TGF-β receptor signalling pathway
HTC	*SPP1,* 25 *IBSP* 25 and *COL10A1* 25	Cartilage development, ossification
preFC	***COL27A1***, ***PLCG2*** and ***WWP2***	RNA splicing and processing
FC	***MMP2*** *, COL1A1* 10 and *COL1A2* 10	Regulation of angiogenesis, ossification
preInfC	***IFI16*** and ***IFI27***	Focal adhesion, regulation of angiogenesis
InfC	***CXCL8***, ***CD74*** and ***GPR183***	The inflammatory response and innate immune response

Part of the marker genes were reported by previous studies and the newly defined marker genes are highlighted in bold.

ECM, extracellular matrix; preFC, prefibrocartilage chondrocyte; preHTC, prehypertrophic chondrocyte; preInfC, pre-inflammatory chondrocyte; TGF, transforming growth factor.

Together, we found four cell populations, that is, preHTC, FC, preInfC and InfC, were dominant in the patients with OA. We then asked the following questions: (1) how these chondrocytes are spatially distributed in the cartilage tissue of both patients with OA and non-OA controls; (2) which chondrocyte populations are OA-critical populations that may contribute to the degeneration of cartilage tissue.

### Spatially resolved transcriptional landscape of human knee cartilage in patients with OA and non-OA controls

**Figure 2:**
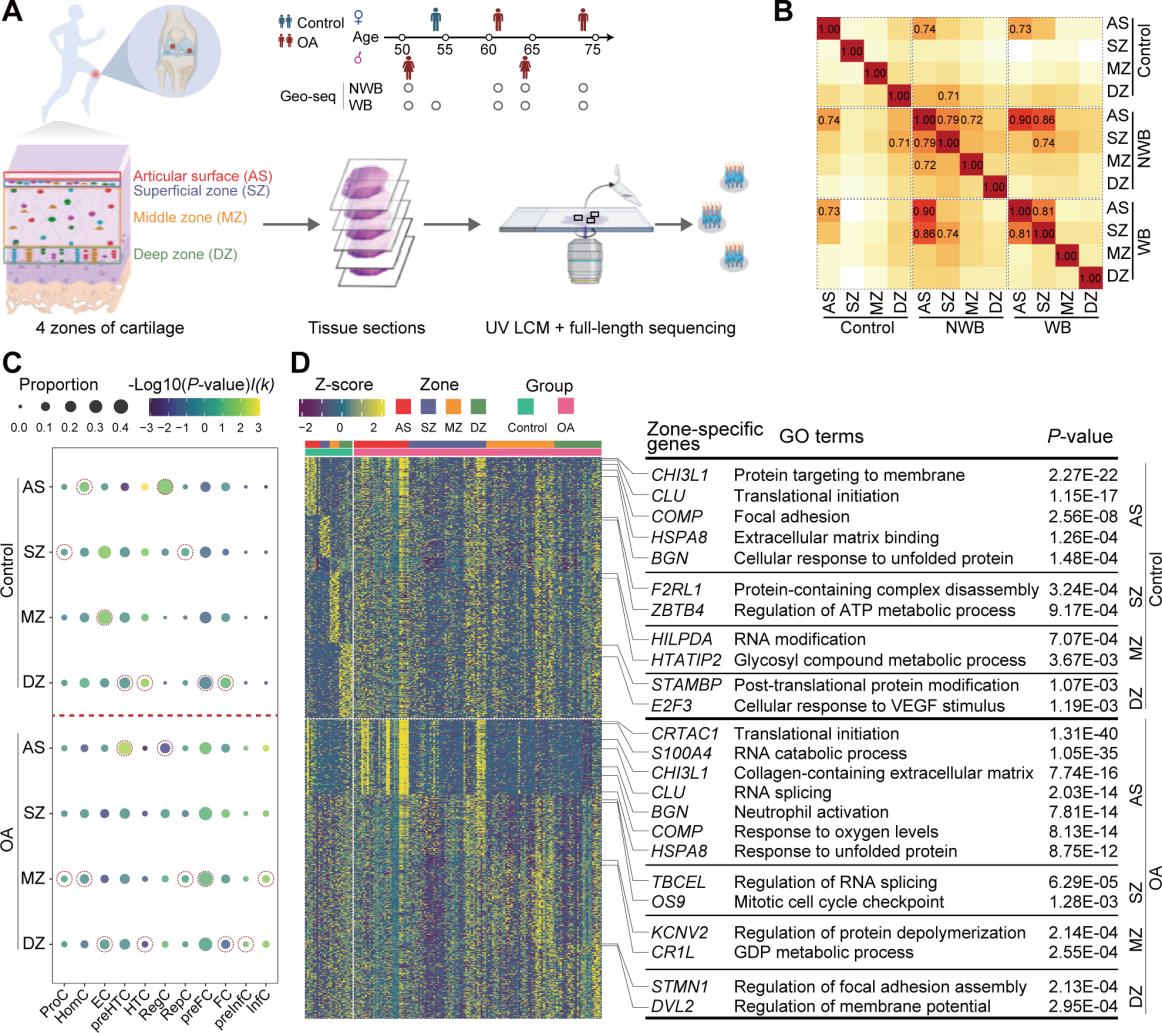
The spatial transcriptomic landscape of human knee cartilage in patients with OA and non-OA controls. (A) Schematic of workflows for transcriptomic profiling of human articular cartilage using Geo-seq (n=5, 2 females and 3 males), including 4 paired WB (53 spots) and NWB (50 spots) and a non-OA control (21 spots). Articular cartilages are separated into four zones, that is, AS, SZ, MZ and DZ based on the organisation and degree of alignment of the collagen fibers. (B) The heatmap showed the transcriptional similarity among four zones from different cartilage conditions. Unlike control samples, AS and SZ zones from OA samples are highly correlated. (C) Cell compositions for each zone mapped spatial landscape by CIBERSORTx. The dot plot showed the cell composition difference between patients with OA and non-OA controls. Gene signature matrices were constructed based on scRNA-seq data. The dot size represents the proportion of each chondrocyte population in a particular zone and the colour represents the −log10 (p value) (permutation t-test). I(k) represents an indicator function, where I(k)=1 if a chondrocyte population is abundant in the patient with OA and I(k)=−1 if a chondrocyte population is abundant in the non-OA controls. The dot which represents the highest cell proportion for each chondrocyte population across four zones is highlighted by dotted circles. (D) The heatmap displays the expression levels of the top 10 zone-specific genes in AS (red), SZ (purple), MZ (orange) and DZ (green) for non-OA control (upper panel) or OA samples (bottom panel). The Gene Ontology terms were enriched by the zone-specific genes of non-OA controls (upper panel) or patients with OA (bottom panel). Zone-specific genes of MZ and DZ were not enriched in any OA-related meaningful pathways. AS, articular surface; DZ, deep zone;Geo-seq, laser microdissection-based RNA sequencing; InfC, inflammatory chondrocyte; LCM, laser capture microdissection; MZ, middle zone; NWB, non-weight-bearing; OA, osteoarthritis; preFC, prefibrocartilage chondrocyte; preHTC, prehypertrophic chondrocyte; preInfC, pre-inflammatory chondrocyte; scRNA-seq, single-cell RNA sequencing; SZ, superficial zone; WB, weight-bearing;

The characterisation of knee articular cartilage changes with different zones—AS, SZ, MZ and DZ—based on the organisation of the tissue and the alignment degree of collagen fibers^27^ (online supplemental figure S4A). To comprehensively explore the spatial landscape of chondrocytes on human knee articular cartilage, we carried out the laser capture microdissection (LCM)^36^ coupled with full-length messenger RNA (mRNA) sequencing (Geo-seq; see ‘Materials and methods’ section) on nine cartilage samples from four patients with OA (ie, paired WB and NWB) and one non-OA control (figure 2A). The details of sample collection from each zone can be found in online supplemental table S5.

To illustrate the relationship among four zones, we computed the cosine similarity between distinct zones across various statues. Notably, we observed that AS and SZ exhibited substantial intercorrelation within both NWB and WB. Furthermore, AS or SZ displayed significant intracorrelation between NWB and WB (figure 2B). In contrast, the four zones from the non-OA controls showcased overall distinct gene expression profiles, indicating their distinctive role in preserving cartilage homeostasis.

Since the Geo-seq data do not achieve single-cell resolution, to comprehensively resolve the distribution of chondrocytes in four different zones, we performed the deconvolution analysis of Geo-seq data using CIBERSORTx^46^ (see ‘Materials and methods’ section). As a result, we observed that preHTCs were mainly located in the AS of OA samples, while in the DZ of non-OA controls; HTCs were mainly located in the DZ of all samples, which were consistent with the aforementioned IHC staining results (figure 1F); InfCs were mainly located in MZ of OA while were absent in non-OA controls (figure 2C). Furthermore, we noted that the compositions of preHTC, HTC, InfC and RegC exhibited great changes between the patient with OA group and the non-OA control group. Specifically, the proportion of preHTC for patients with OA and non-OA controls in AS was 30.3% and 7.74%, respectively (p=4.76e-3 using permutation t-test); the proportion of HTC in DZ was 3.01% and 8.51% (p=9.62e-3); the proportion of RegC in AS was 14.9% and 41.7% (p=0.027) and the proportion of InfC in MZ was 6.07% and 0% (p=0.024).

In contrast, we noted the proportions of preFC in the AS and SZ exhibited substantial variations between WB and NWB (online supplemental figure S4B). In particular, the cell proportions of preFC in the AS were 18.8% and 31.7%, respectively (p=0.013) while in the SZ were 20.4% and 41.7%, respectively (p=4.25e-5).

### The zone-specific genes of human knee cartilage in patients with OA and non-OA controls

Next, we sought to comprehensively examine the four zone-specific genes for the human knee articular cartilage. To this end, we performed zone-specific gene detection using DESeq2^47^ (see ‘Materials and methods’ section; log2FC≥1, and adjusted p<0.05). As a result, we identified 561, 190, 245 and 737 zone-specific genes for AS, SZ, MZ and DZ of non-OA controls, respectively; 2777, 694, 1418 and 691 zone-specific genes for AS, SZ, MZ and DZ of patients with OA, respectively (online supplemental table S6).

In particular, we observed that the zone-specific genes showed distinct expression patterns between patients with OA and non-OA controls (figure 2D) and were enriched in biologically meaningful Gene Ontology (GO) terms (online supplemental table S7). In particular, glycoprotein-related genes, that is, *COMP, CLU* and *BGN* were commonly highly expressed in AS of both OA and non-OA controls. In addition, the protein folding-related genes (eg, *HSPA8*, *HSPB8*) were also highly expressed in AS of both OA and non-OA controls, and were commonly enriched in the cellular response to unfolded protein pathway, suggesting that the chondrocytes in AS are essential for ECM formation. However, chondrocyte proliferation-associated genes such as *S100A4*, and *CDKN1A*, and hypoxia-associated genes such as *VEGFA*, and *EGR1*, *PRG4* were uniquely highly expressed in AS of patients with OA, suggesting a higher proliferative activity and stress in OA cartilage samples. *CRTAC1*, a candidate biomarker for OA was also found to be highly expressed in AS of patients with OA. Moreover, the SZ-specific genes of non-OA controls contributed to energy metabolism, whereas these zone-specific genes of patients with OA were involved in cell cycle-associated pathways, suggesting that the chondrocytes in the SZ entered a proliferative cell cycle to compensate for damage in the AS. Finally, MZ-specific and DZ-specific markers of both patients with OA and non-OA controls were not enriched in any biologically meaningful pathways. Therefore, AS and SZ are molecularly unique among all zones, whereas MZ and DZ are more ubiquitous. These results are consistent with a previous zone-specific gene expression analysis of articular cartilage.^48^

Next, we examined the zone-specific genes for WB and NWB. We identified 359, 704, 1847 and 230 genes for AS, SZ, MZ and DZ of NWB, respectively; 3539, 514, 729 and 782 genes for AS, SZ, MZ and DZ of WB, respectively (online supplemental table S6). Specifically, we found that AS-specific genes from WB and NWB were enriched in similar pathways, including protein folding and hypoxia-related pathways (online supplemental figure S4C). Besides, AS-specific genes from WB samples were further involved in apoptotic signalling pathways, with highly expressed *MAPK9*, *IFI6* and *BCL10*, etc. Moreover, SZ-specific genes from NWB were mainly enriched in cell cycle-associated pathways, while SZ-specific genes from WB were mainly enriched in autophagy-related pathways (online supplemental table S7). Similarly, we observed that MZ-specific and DZ-specific markers of both NWB and WB samples were not enriched in any biologically meaningful pathways.

### Cell population InfC is uniquely identified in patients with OA

**Figure 3:**
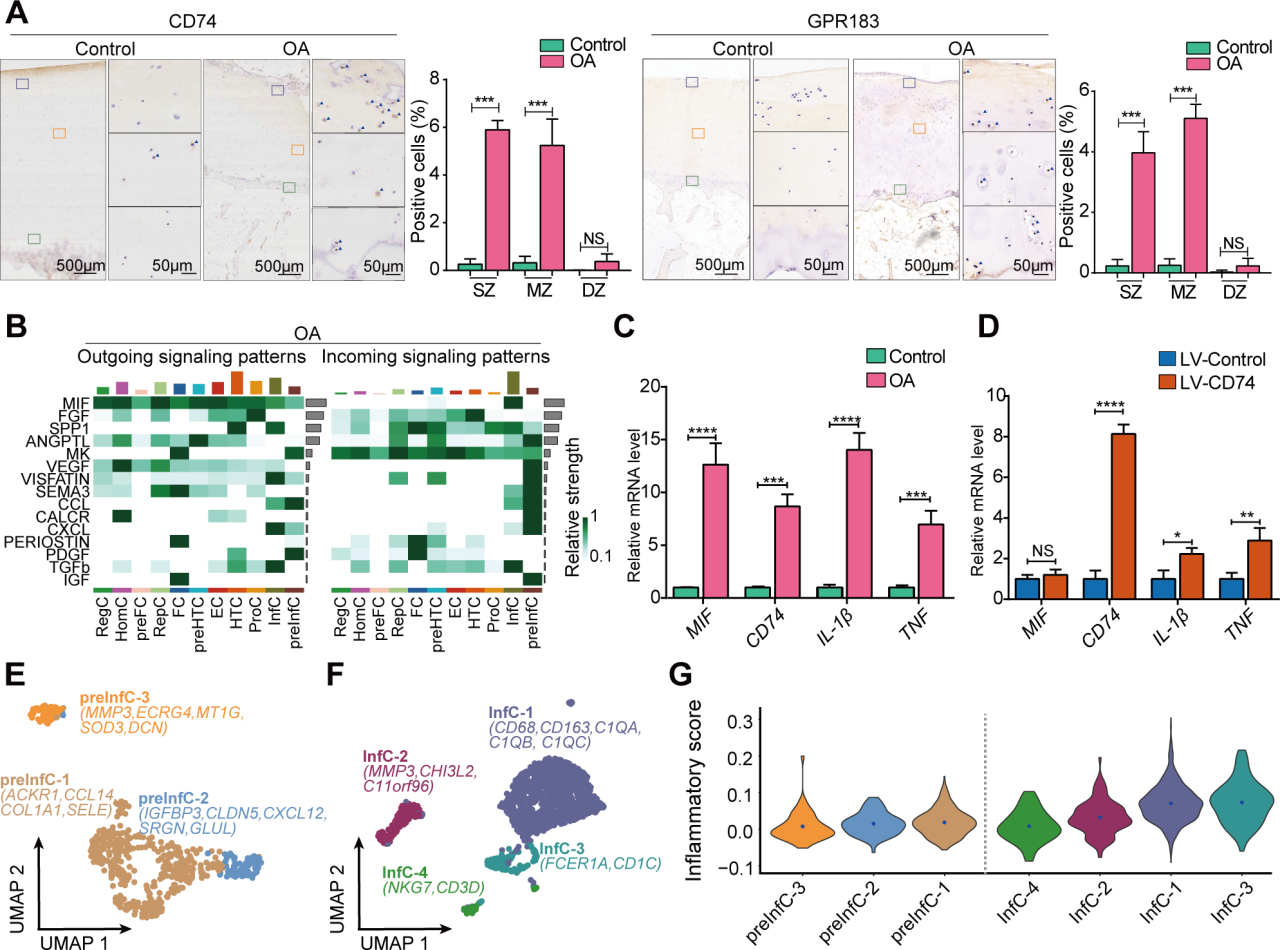
The roles of the InfC and preInfC populations in patients with OA and non-OA controls. (A) The marker genes (ie, CD74 and GPR183) for the InfC population are validated by IHC. The quantification of positive cells from different zones (ie, SZ, MZ and DZ) is displayed by bar plots (replicates n=6), where the AS and SZ were hardly distinguished during IHC staining, therefore, the AS and SZ in Geo-seq results were merged together to only present as SZ in IHC staining. (B) A heatmap shows cell-cell communications in patients with OA. The grey bar on the y-axis denotes the relative strength of the predicted signalling pathway in the overall network while the colour bar on the x-axis represents the relative contribution of the chondrocyte population to the overall signalling milieu (top x-axis) for patients with OA. MIF was ranked as the strongest signalling pathway that interacted with chondrocyte populations in OA samples. (C) Quantitative real-time PCR was performed to measure the relative mRNA levels of MIF, CD74, IL-1β and TNF in patients with OA and controls (replicates, n=6). (D) Quantitative real-time PCR was performed to measure the relative mRNA levels of CD74, MIF, IL-1β and TNF after CD74 overexpressing with LV (replicates, n=6). (E) The visualisation of the subpopulation of preInfC using UMAP projections, that is, preInfC-1 (brown), preInfC-2 (blue) and preInfC-3 (orange), where a total of 533 chondrocytes were analysed together by the integrative analysis of patients with OA. The marker genes of each subpopulation were also annotated. (F) The visualisation of the subpopulation of InfCs using UMAP projections, that is, InfC-1 (cyan), InfC-2 (crimson), InfC-3 (dark green) and InfC-4 (green), where a total of 1499 chondrocytes were analysed together by the integrative analysis of patients with OA. (G) The violin plot shows the inflammatory score for the subpopulations of preInfC and InfC. The inflammatory scores for each chondrocyte were calculated through the average expression of a predefined inflammatory-associated gene set. The inflammatory scores were gradually increasing from preInfC to InfC. AS, articular surface; DZ, deep zone; IHC, immunohistochemistry; IL, interleukin; InfC, inflammatory chondrocyte; MIF, macrophage migration inhibitory factor; mRNA, messenger RNA; MZ, middle zone; NS, no significant; OA, osteoarthritis; preInfC, pre-inflammatory chondrocyte; SZ, superficial zone; TNF, tumour necrosis factor; UMAP, uniform manifold approximation and projection. *p<0.05, **p<0.01, ***p<0.001, ****p<0.0001.

With the results from the scRNA-seq and Geo-seq data, we observed the InfC specifically expressed *CD74*, *GPR183* and *CXCL8* marker genes, and spatially distributed in MZ of patients with OA (figure 2C). We performed both CD74 and GPR183 IHC staining on patients with OA and non-OA controls to validate their expression levels. As a result, we noted that these genes were significantly expressed in SZ and MZ of patients with OA (figure 3A). To examine the origin of such novel chondrocytes, we performed trajectory inference^49^ to infer the pseudotime along with chondrocyte differentiation (see ‘Materials and methods’ section). We found that preInfCs and InfCs were located in the termination point of lineage (online supplemental figure S1B), indicating that InfCs may be differentiated from the preInfCs.

To further confirm the differentiation trajectory of InfCs, we statistically grouped the chondrocytes into four clusters based on transcriptional changes along the pseudotime axis (online supplemental figure S5A). Specifically, cluster 1 exhibited significant expression of marker genes for ProC (eg, *HMGA1*, *BMP2*, etc) and marker genes for HomC (eg, *DDIT3*, *HSPA6*, etc) as well as cell proliferation and cell cycle-associated genes such as *CDKN1A* and *G0S2*. Cluster 2 displayed upregulation of marker genes associated with RegC, HTC, RepC and EC, primarily linked to ECM organisation, cartilage development and tissue homeostasis. In contrast, the marker genes for preHTC (e.g. *CRTAC1* and *ABI3BP*), and preFC (e.g. *COL27A1*), were upregulated in cluster 3, which were associated with ECM organisation, glycosaminoglycan and ion channels. Finally, the markers of preInfC (e.g. *IFI27*), InfC (e.g. *CD74* and *GPR183*) and FC (e.g. *COL1A1*) were highly expressed in cluster 4, which were enriched in angiogenesis and inflammatory response pathways, such as neutrophil activation and leucocyte migration (online supplemental figure S5B). Overall, these results further confirmed that the inflammatory signature was mainly present in the chondrocytes that were located at the termination of the differentiation process.

### InfC-induced MIF-CD74 signalling may be an important regulator in the pathogenesis of OA

To systematically investigate the cell-cell communications of chondrocytes, we then performed ligand-receptor (L-R) interaction analyses using CellChat^50^ (see ‘Materials and methods’ section). This analysis unveiled 15 potential L-R interactions relevant to patients with OA (figure 3B) and identified 11 potential L-R interactions linked to non-OA controls (online supplemental figure S6A). Additionally, our investigation revealed that multiple inflammatory signalling pathways were only involved in the patients with OA samples. In particular, preInfC and InfC were identified as both sender and receptor through chemokine signalling pathways, for example, CCL and CXCL (figure 3B). Notably, among all these L-R interactions, we found MIF was the major relative strength in outgoing signalling patterns in patients with OA, which has been reported in previous studies.^51 52^ The analysis of L-R interactions revealed that MIF triggered pro-inflammatory effects via two distinct pairs, MIF-CD74+CD44 and MIF-CD74+CXCR4, particularly observed in InfC and communicated with other chondrocyte populations (online supplemental figure S6B). Previous studies have shown elevated MIF protein levels in OA cartilage, and *MIF* deletion has been associated with reduced OA severity.^52^ Notably, our findings emphasised that the interaction of MIF with CD74 expressed by the InfC population may contribute to the pathogenesis of OA. Furthermore, we observed the chondrocyte populations in non-OA controls also engaged in the MIF pathway, with preHTC acting as the primary receptor population (online supplemental figure S6A). However, this interaction used a distinct L-R pair, MIF-ACKR3 (online supplemental figure S6C). The activation of the PI3K-Akt pathway by MIF-ACKR3 further supported chondrocyte proliferation and hindered hypertrophic differentiation,^53 54^ reinforcing the pro-inflammatory role of the InfC and preInfC populations in the cellular milieu of cartilage tissue in patients with OA.

Moreover, for additional confirmation, we pursued two distinct validation approaches: we gauged the relative mRNA levels of marker genes for InfCs using quantitative real-time PCR (qRT-PCR), revealing a significant upregulation of *MIF* and *CD74* in OA chondrocytes (figure 3C). Subsequently, we investigated the biological function of *CD74*+InfCs by overexpressing *CD74* in the C28/I2 human chondrocyte cell line (see ‘Materials and methods’ section). Interestingly, this manipulation led to an increase in the gene expressions of renowned pro-inflammatory cytokines *IL-1β* and *TNF* 55 56 (figure 3D).

### The subpopulations of preInfC and InfC demonstrate diverse inflammatory score

Next, we sought to explore both InfC-specific and preInfC-specific gene expression changes that may further explain the role of inflammatory-associated chondrocytes in OA. To do so, we performed the subclustering analysis, delving deeper into the compositions of both populations (see ‘Materials and methods’ section). Notably, three distinct subpopulations of preInfC were identified (figure 3E). The preInfC-1 cells exhibited elevated expression of inflammatory chemokines-associated genes (eg, *CCL14* and *ACKR1*) and FC markers like *COL1A1*, suggesting an intermediary state between FC and InfC. The preInfC-2 cells demonstrated heightened expression of *CXCL12*, potentially linked to chondrocyte apoptosis.^57^ The preInfC-3 cells exhibited high expression levels of *MMP3*, *ECRG4* and *DCN*, indicative of cartilage degradation. Furthermore, we identified four hypothetical subpopulations of InfC (figure 3F). The InfC-1 cells highly expressed classic macrophage markers *CD68*, *CD163* and several members of complement pathway-related genes (ie, *C1QA, C1QB* and *C1QC*). The InfC-2 cells highly expressed *MMP3*, *CHI3L2* and *C11orf96*. Finally, the InfC-3 and InfC-4 highly expressed dendritic cell markers (eg, *FCER1A* and *CD1C*) and T/NK cell markers (eg, *NKG7* and *CD3D*), respectively.

Additionally, we sought to investigate the potential contribution to inflammation for each subpopulation. To this end, we initially established an inflammatory score for individual chondrocytes, determined by the expression of a curated set of genes related to inflammation. Subsequently, these scores served as indicators to evaluate the inflammatory profile for each subpopulation^58^ (see ‘Materials and methods’ section). In examining the subpopulations of preInfC, we observed that the inflammatory score of preInfC-1 (mediate value=0.016) and preInfC-2 (mediate value=0.012) was higher than that of preInfC-3 (mediate value=−0.002). Furthermore, all InfC subpopulations exhibited elevated inflammatory scores (figure 3G), that is, mediate value=0.067 for InfC-1; 0.028 for InfC-2; 0.074 for InfC-3 and 0.005 for InfC-4. Lastly, we noted a substantial correlation between the inflammatory score of chondrocytes and the pseudotime depicted in online supplemental figure S1B (Spearman’s r=0.527, p<2.2e-16). This observation provides further evidence supporting the potential for chondrogenic differentiation from preInfC to InfC.

### The preHTC displays the major changes between patients with OA and non-OA controls

To further quantify the compositional differences between patients with OA and the non-OA controls within the shared chondrocyte populations, we performed a cell population-specific composition shift analysis using Cacoa^59^ (see ‘Materials and methods’ section). As a result, we observed that the most significant composition change between patients with OA and the non-OA controls was in preHTC (20.4% vs 8.9%, p=2.0e-4), followed by RepC (8.46% vs 5.54%, p=2.0e-4, online supplemental figure S7A).

**Figure 4:**
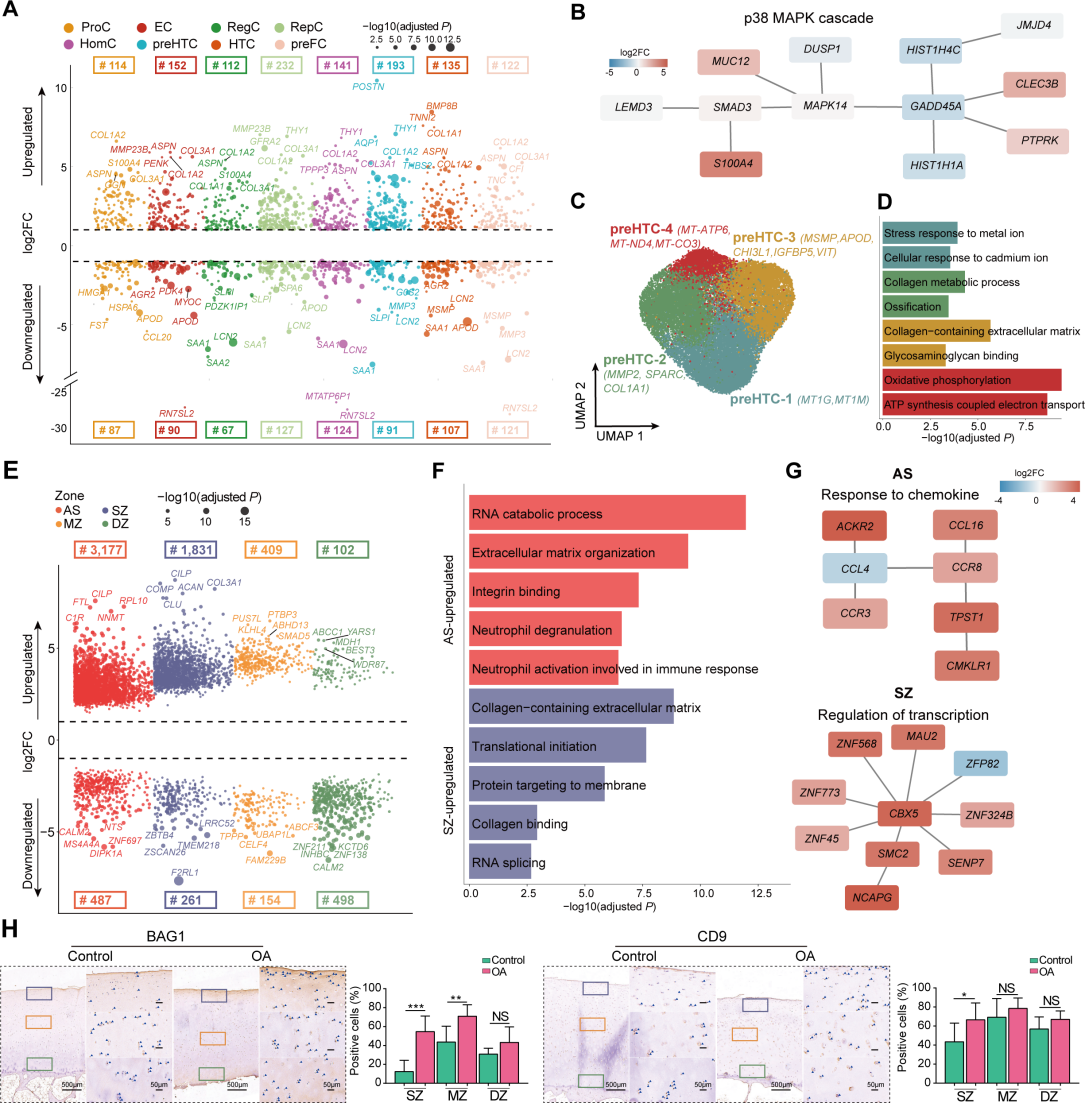
The OA-associated changes between patients with OA and non-OA controls. (A) The Manhattan-like plot demonstrates the population-specific DE genes between patients with OA and non-OA controls. The numbers on the top panel denote the number of DE genes for each population. The dot size represents the value of −log10 (adjusted p). (B) The subnetworks identified by PhenomeExpress. The DE genes of preHTC were enriched in a pMAPK38 cascade subnetwork. (C) The visualisation of subpopulations of preHTC using UMAP projections, that is, preHTC-1 (blue), preHTC-2 (green), preHTC-3 (brown) and preHTC-4 (red), where a total of 25 778 chondrocytes were analysed by the integrative analysis of all 19 samples. (D) The bar plot shows the significant GO terms for each subpopulation. (E) The Manhattan-like plot shows the zone-specific DE genes between patients with OA and non-OA controls, that is, AS (red), SZ (yellow), MZ (blue) and DZ (green). The numbers on the top panel or bottom panel represent the number of significant upregulated or downregulated DE genes for each zone. (F) The bar plot shows the significant GO terms that were enriched by the zone-specific DE genes. Notably, the MZ-specific and DZ-specific DE genes were not enriched in any biologically meaningful pathways. (G) The subnetworks identified by PhenomeExpress. The DE genes of AS were enriched in response to the chemokine subnetwork and those of SZ were enriched in the regulation of the transcription subnetwork. (H) The top-ranked DE gene of AS or SZ between patients with OA and non-OA controls, BAG1, was validated by IHC staining. (I) The top-ranked DE gene of AS or SZ between patients with OA and non-OA controls, CD9, was validated by IHC staining. The quantification of positive cells from different zones (ie, SZ, MZ and DZ) is displayed by bar plots (replicates n=6), where the AS and SZ were hardly distinguished during IHC staining, therefore, the AS and SZ in Geo-seq results were merged together to only present as SZ in IHC staining. The scale bar: left, 500 μm; right, 50 μm. AS, articular surface; DZ, deep zone; GO, Gene Ontology; IHC, immunohistochemistry; InfC, inflammatory chondrocyte; MZ, middle zone; NS, no significant; OA, osteoarthritis; preInfC, pre-inflammatory chondrocyte; preFC, prefibrocartilage chondrocyte; preHTC, prehypertrophic chondrocyte; SZ, superficial zone; UMAP, uniform manifold approximation and projection. *p<0.05, **p<0.01, ***p<0.001.

Next, to identify OA-associated genes, we performed differential expression (DE) analysis using DESeq2 (see ‘Materials and methods’ section) on pseudobulk data,^60^ that is, aggregating (ie, summation) gene expression raw counts of all chondrocytes within one population. Among the DE genes across all shared cell populations ( log2FC≥1 , and adjusted p<0.05; online supplemental table S8), we identified a total of 23 upregulated common DE genes (online supplemental figure S7B), which were associated with collagen-containing ECM (eg, *COL3A1, COL1A2* and *COL6A3*), and matrix-degrading enzymes associated genes (eg, *ADAMTS6* and *HTRA1*). Notably, we observed that the upregulated DE genes across all chondrocyte populations participated in analogous pathways (online supplemental figure S7C), indicating a shared transcriptomic characteristic among OA chondrocytes—specifically, dysregulated ECM metabolism, cell proliferation and differentiation. Broadly, preHTC and RepC detected a greater number of DE genes between patients with OA and non-OA controls (figure 4A). Specifically, preHTC uniquely identified the osteogenic-related genes such as *POSTN, CCN4* and *TGFB3* (online supplemental figure S7B), which enriched in bone development pathway. In contrast, the other cell population’s unique DE genes were not enriched in any significant pathways.

Additionally, we performed gene-gene network analysis (see ‘Materials and methods’ section) that combines information from DE analysis with protein-protein interaction networks to identify statistically OA-associated subnetworks. This analysis further confirmed the connection of preHTC DE genes to the p38 MAPK cascade pathway (figure 4B), which potentially contributed to the apoptosis of chondrocytes.^61^

### The preHTC exhibits the greatest expression changes between WB and NWB

The aforementioned results demonstrated that preHTC exhibited the major compositional changes between patients with OA and non-OA controls. Therefore, we then asked how the gene expression changes in preHTC between WB and NWB. To do so, we first performed expression shift analysis using Cacoa. Notably, we observed that the cell composition between WB and NWB exhibited small distinctions (online supplemental figure S8A). Moreover, a few numbers of DE genes (<10 genes) between WB and NWB were detected across most cell populations (an adjusted p of 5% and $\left|log2FC\right|\geq 0.5$ ), suggesting minor gene expression variations between WB and NWB (online supplemental figure S8B).

In contrast, the cell populations with the most three detected DE genes were EC, preHTC and RepC, with a total of 20, 35 and 34 DE genes, respectively. Specifically, we noted that these chondrocytes were highly expressed ECM-related genes, such as *TGFBI*, *FN1*, *COL1A2*, *COL1A1* and *LUM*. Meanwhile, preHTC was uniquely expressed by the matrix-degrading enzymes *ADAMTS2* and MMP inhibitors *TIMP3*, along with the inflammatory-associated gene *THY1* (online supplemental figure S8B). These observations are consistent with the previous studies.^62 63^

The accumulating evidence suggests that preHTC may play a critical role in OA pathogenesis. Therefore, to further investigate the cellular heterogeneity of preHTC, we performed the subclustering analysis, resulting in four putative subpopulations (figure 4C and online supplemental figure S8C). Specifically, the preHTC-1 highly expressed the marker genes that related to metal and cadmium ions, such as *MT1G* and *MT1M*. This subpopulation was also reported as the metallothionein chondrocytes (MTC) by previous studies.^25^ The preHTC-2 was abundant in patients with OA and highly enriched in the ossification pathway (p=1.36e-5; *COL1A1*, *MMP2* and *SPARC*). In contrast, the preHTC-3 highly expressed *APOD* and *CHI3L1*, potentially play a role in cartilage protection from ECM degradation.^64^ The preHTC-4 was abundant in patients with OA and highly expressed in the mitochondrial genes, such as *MT-ATP6*, *MT-ND4* and *MT-CO3*, which may further contribute to cartilage degeneration^65^ (figure 4D).

### The AS and SZ exhibit the most significant alterations in gene expression

To investigate the zone-specific changes in function, we performed the zone-specific DE analysis of Geo-seq data using DESeq2 (see ‘Materials and methods’ section). As a result, we identified 3177, 1831, 409 and 102 upregulated DE genes ( log2FC≥1 , and adjusted p<0.05) for AS, SZ, MZ and DZ, respectively, when comparing between patients with OA and non-OA controls (figure 4; online supplemental table S9). In particular, we found that ECM-associated genes (eg, *FN1, COL1A1* and *COMP*) were consistently expressed in both the AS and SZ of OA samples. Meanwhile, the upregulated genes in AS were enriched in immune response-related pathways, for example, neutrophil activation involved in immune response (*IL1R2*, *MAPK14* and *CD9*). The upregulated genes in SZ were enriched in transcription and protein synthesis-related pathways (figure 4; online supplemental table S10). The gene-gene network analysis also demonstrated the response to the chemokine subnetwork in AS and the regulation of the transcription subnetwork in SZ significantly, further confirming the enhanced inflammatory response and transcription in OA (figure 4G). In contrast, despite the detection of numerous upregulated DE genes in the MZ and DZ between patients with OA and non-OA controls, these genes did not enrich meaningful pathways. Furthermore, we identified 478, 261, 154 and 498 downregulated DE genes between patients with OA and non-OA controls for the AS, SZ, MZ and DZ, respectively (figure 4; online supplemental table S9). However, those genes were not enriched in any biologically meaningful pathways.

Since the sample size issue of the non-OA controls in Geo-seq data potentially introduced unwanted variations, we performed three additional lines of validation. We first curated a total of 2886 OA-associated genes from PubMed with a given search criterion (see ‘Materials and methods’ section). As a result, we observed that the upregulated DE genes for AS and DZ were enriched in the given gene set in a range of proportions (online supplemental figure S9A). We next collected a total of 6552 OA-associated pathways with a given search criterion (see ‘Materials and methods’ section). As a result, we found that 60.2% and 52.0% of pathways overlapped with DE genes from AS and SZ, respectively (online supplemental figure S9B). Finally, we conducted BAG1 and CD9 IHC staining on patients with OA and non-OA controls. Notably, protein folding-related gene BAG1 and immune response regulator CD9 were highly expressed in SZ (figure 4H).

In addition, we also performed the DE analysis between WB and NWB, resulting in a total of 628, 1734, 590 and 2072 upregulated DE genes ( log2FC≥1 , and adjusted p<0.05) for the AS, SZ, MZ and DZ, respectively (online supplemental figure S10A; online supplemental table S11), and we identified a total of 15, 914, 1156 and 362 downregulated DE genes ( log2FC≤-1 , and adjusted p<0.05) for the AS, SZ, MZ and DZ, respectively. Specifically, we observed that the minimum number of DE genes were detected in AS between WB and NWB, suggesting the small gene expression alterations between WB and NWB. The highest number of DE genes were identified in SZ, which were enriched in protein synthesis and ECM-related pathways (online supplemental figure S10B online supplemental table S12). Notably, a large number of DE genes were detected in the MZ and DZ, however, these genes were not enriched in biologically meaningful pathways.

### Cellular composition and expression signature of preHTC

To further confirm the critical role of preHTC in large-scale population studies, we estimate the cell composition of bulk samples by performing deconvolution analysis on two publicly available bulk RNA-seq datasets using CIBERSORTx.^46^ Here, the cell population-specific gene signature matrices were curated from the scRNA-seq data in this study (see ‘Materials and methods’ section).

**Figure 5:**
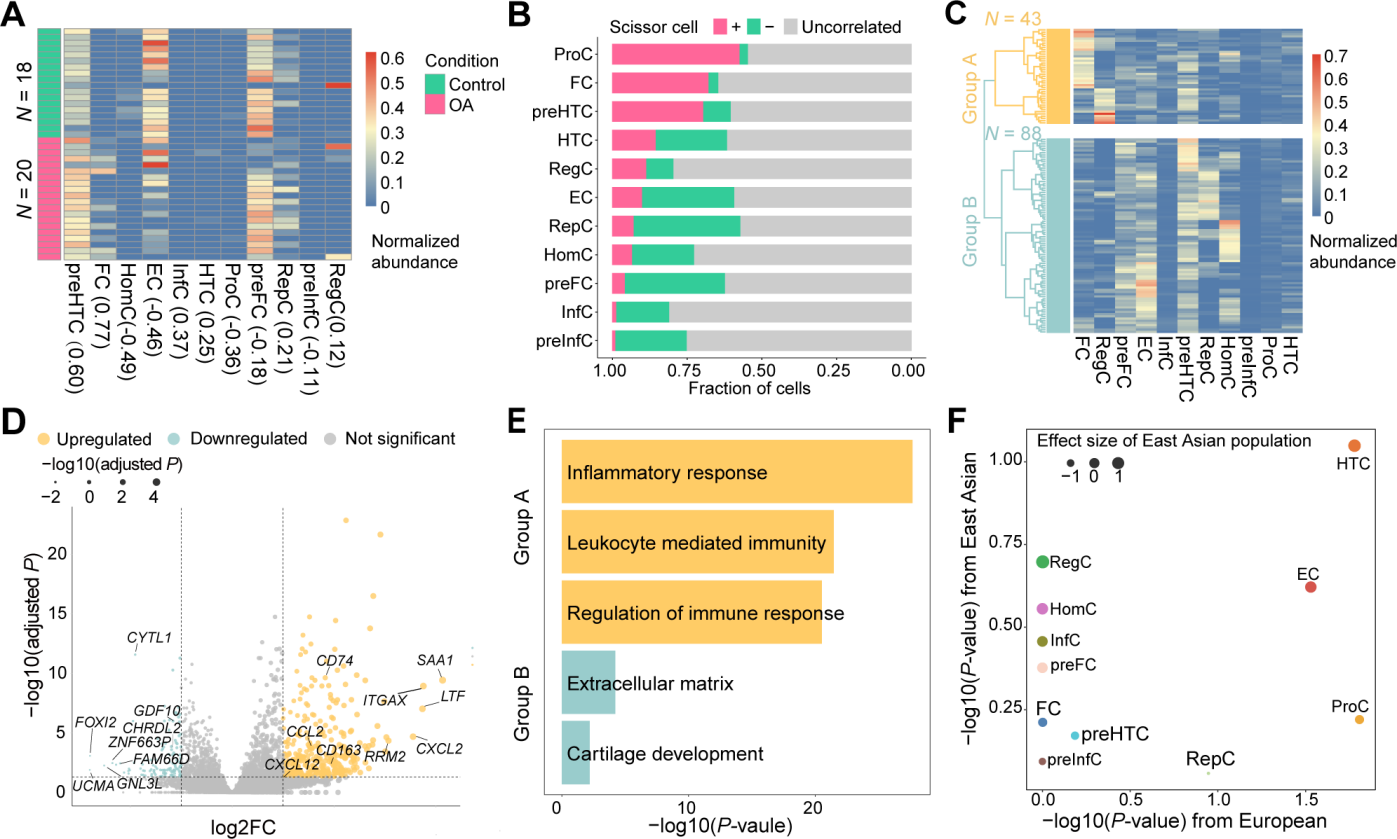
The OA-critical cell populations in bulk RNA-seq data and GWAS associations. (A) The heatmap shows cell compositions of 18 healthy controls and 20 patients with OA from GSE114007. The values in brackets represent the Spearman’s correlation (r) between patients with OA and non-OA controls. FC and preHTC are highly correlated with OA status. (B) The bar plot depicts the fraction of chondrocytes that are related to OA, where Scissor+ denotes the positively correlated (pink), Scissor− is negatively correlated (green), and the others are uncorrelated chondrocytes (grey). The top three populations, ProC, FC and preHTC are positively correlated with OA status. (C) The heatmap illustrates the two subtypes of 131 OA samples identified by hierarchical cluster with ward.D2 parameter. 131 samples are clustered into 2 subtypes: group A (n=43): an inflammation-related subtype with less fraction of preFCs and group B (n= 88): a non-inflammatory-related subtype with more fraction of preFCs. (D) The volcano plot shows the differentially expressed genes between group A versus group B. (E) The significant Gene Ontology terms were enriched by the upregulated/downregulated DE genes. The upregulated DE genes in group A (inflammation-related subtype) were mainly involved in inflammatory and immune response-associated pathways, while group B (non-inflammatory-related subtype) upregulated DE genes were mainly involved in chondrocyte proliferation pathways. (F) The scatter plot shows the correlation between the cell population contribution to OA as determined from GWAS of individuals with Asian ancestry (n=1979) and the corresponding contribution derived from GWAS of individuals with European ancestry (n=406 241). HTC population is significantly associated with OA in individuals with European ancestry and has the smallest p value with East Asian ancestry. GWAS, genome-wide association studies; InfC, inflammatory chondrocyte; OA, osteoarthritis; preFC, prefibrocartilage chondrocyte; preHTC, prehypertrophic chondrocyte.

In the first bulk RNA-seq data consisting of 38 samples (20 patients with OA and 18 controls), we observed that InfC and FC were only presented in patients with OA (the proportion: 0% vs 0.11% for non-OA vs OA; p=0.048 for InfC, and 0% vs 8.75%; p=8.29e-5 for FC). preHTC (20.0% vs 32.1%; p=4.88e-5) was significantly more abundant in patients with OA samples. In contrast, HomC was significantly more abundant in non-OA controls (2.41% vs 0.046%; p=5.7e-4, figure 5A). This result was consistent with the cell compositions in the integrative analysis of scRNA-seq data and Geo-seq data.

To identify cell subpopulations from single-cell data that the transcriptional patterns are associated with OA status, we applied *Scissor* 66 to such bulk RNA-seq data. We classified chondrocytes that were positively associated (Scissor^+^ cells), negatively associated (Scissor^−^ cells) and uncorrelated (background cells) with OA pathology (see ‘Materials and methods’ section). As a result, we found that ProC was significantly associated with OA (42.6% Scissor^+^ cells and 2.82% Scissor^−^ cells), followed by FC (32.2% Scissor^+^ cells and 3.28% Scissor^−^ cells) and preHTC (30.5% Scissor^+^ cells and 9.19% Scissor^−^ cells; figure 5B), supporting the association of preHTC with OA status.

The second bulk RNA-seq data, comprising eight damaged distal medial condyles (DMC, equivalent to WB), and eight matched intact posterior lateral condyles (PLC, equivalent to NWB) served as internal controls (see ‘Materials and methods’ section). Through deconvolution analysis, it was revealed that preFC was predominant in PLC, constituting 30.5% compared with 18.0% in DMC (p=6.84e-3, online supplemental figure S11A). The absence of preFC in DMC aligned with the cell compositions observed in the Geo-seq data, comparing WB and NWB. Scissor analysis also demonstrated that the preHTC was significantly associated with DMC (24.0% Scissor^+^ cells and 0.48% Scissor^−^ cells; online supplemental figure S11B).

To further explore the role of preHTC, we applied Scissor to the integrative analysis of four subpopulations and the two bulk RNA-seq datasets. Notably, we observed that preHTC-2 was strongly associated with either OA status (21.8% Scissor^+^ cells for preHTC-1, 63.0% for preHTC-2, 15.6% for preHTC-3 and 21.7% for preHTC-4) (online supplemental figure S11C) or DMC (18.7% Scissor^+^ cells for preHTC-1, 74.5% for preHTC-2, 6.2% for preHTC-3 and 29.7% for preHTC-4) (online supplemental figure S11D). These results highlight the critical role of the preHTC-2 subpopulation (*MMP2* and *COL1A1* as marker genes) in the pathogenesis of OA.^67^

Finally, we evaluated the predictive capability of OA status using the cell population-specific DE genes. To do so, we employed a logistic regression-based prediction model on the first bulk RNA-seq dataset. With the leave-one-out cross-validation (LOOCV) strategy, we found the DE genes of preHTC exhibited superior performance in predicting OA status (area under the curve (AUC)=0.99; online supplemental figure S12).

### The preFC is the main contributor to subtyping patients with OA

According to the aforementioned results, the patients with OA displayed diverse phenotypes. Therefore, to investigate the subtypes of patients with OA, we applied deconvolution analysis to two bulk RNA-seq datasets.^15 68^ The first bulk RNA-seq dataset contains 131 patients with OA. We then performed a hierarchical clustering analysis on the deconvolution results, resulting in two major subtypes (figure 5C), namely group A (n=43) and group B (n=88). The main differences in cell compositions between group A and group B were the preFC (7.39% vs 15.5%, p=3.17e-9) and the FC (23.6% vs 5.2%; p=3.96e-13). Furthermore, we performed the DE analysis between group A and group B. As a result, we finally identified a total of 457 DE genes (|log2FC|>1 and adjusted p<0.05), including 342 upregulated genes and 115 downregulated genes (figure 5; online supplemental table S13).

Subsequently, we performed an enrichment analysis of the DE genes, finding the upregulated DE genes in group A were strongly enriched in immune-related signalling pathways (expressed: *CXCL12*, *CD163, CCL2* and *CD74*), while the downregulated DE genes were enriched in chondrocyte proliferation-related signalling pathways (expressed: *GDF10, CYTL1* and *CHRDL2*; figure 5E). In addition, this bulk RNA-seq data also contained the joint space narrowing (JSN) score,^69^ which measured the grade of knee OA severity for each patient. We found that the JSN score was significantly higher in group A than in group B (median scores for group A vs group B were 3.75 vs 3.02, p=0.01, Wilcoxon rank-sum test), indicating more severe cartilage lesions in group A. The proportion of preFC in each patient was also negatively correlated with their JSN score in 131 patients with OA (Spearman’s r=−0.239, p=5.93e-3). The stratification of patients with OA was largely consistent with the result in the Research Arthritis and Articular Cartilage (RAAK) study.^68^ Our result suggested that the preFC population was a main contributor to subtyping patients with OA.

The second bulk RNA-seq dataset contains 20 patients with OA. We then performed a hierarchical clustering analysis on the deconvolution results, resulting in two distinct subtypes of OA (online supplemental figure S13A), namely group A (n=7) and group B (n=13). The most different cell composition between group A and group B was preFC (proportion: 7.91% vs 37.1%; p=1.29e-5). Furthermore, we performed DE analysis between group A and group B, detecting a total of 3789 DE genes (|log2FC|>1 and adjusted p<0.05), of which 2125 were upregulated genes in group A and 1664 were downregulated genes (online supplemental figure S13B; online supplemental table S14). In addition, we performed the enrichment analysis, finding the DE genes in group A were enriched in immune-related signalling pathways (expressed: *CXCL12*, *CCL2* and *CD74*), while the DE genes from group B were enriched in chondrocyte proliferation-related signalling pathways (expressed: *SOX5* and *BMP6,* online supplemental figure S13C).

### The HTC genetically relates the GWAS variants in OA

To systematically investigate the cell population-specific GWAS variants that potentially contribute to the pathogenesis of OA, we performed the integrative analysis on scRNA-seq data and GWAS summary data using CELLECT^70^ (see ‘Materials and methods’ section). Since our scRNA-seq samples were all from East Asia (ie, China), we stratified the UK Biobank data to include only individuals of East Asian ancestry. There was a total of 1979 East Asian ancestry participants (1172 individuals with knee OA and 807 controls) for whom we obtained GWAS summary statistics (online supplemental figure S14A) using GMMAT^71^ (see ‘Materials and methods’ section). Next, we used CELLECT to determine the potential genetically relevant chondrocyte populations (see ‘Materials and methods’ section). Specifically, we observed that the strongest cell population-specific association was with HTC (p=0.089) for East Asian populations (figure 5F), reinforcing the notion that HTC may be an OA-critical chondrocyte population.

Furthermore, we performed a similar analysis on European ancestry. Specifically, GWAS summary statistics for knee pain associations from the UK Biobank,^72^ including 406 241 individuals of European ancestry (21 684 knee OA samples and 384 458 controls) were obtained from Neale’s laboratory^73^ (online supplemental figure S14B). We then integrated this GWAS summary statistics with the scRNA-seq data obtained from European samples.^74^ We observed that both ProC (p=0.016) and HTC (p=0.017) were strongly associated with OA status (figure 5F).

Taken together, we systematically investigated the relationship between OA-associated GWAS variants and chondrocyte populations of knee articular cartilage, finding HTC to be the most relevant chondrocyte population in both East Asian and European populations. Our results provided new insight into the genetic architecture of OA and potential therapeutic targets for OA-modifying drugs.

## Discussion

In the knee articular cartilage tissue, chondrocyte resides in a three-dimensional spatial structure, exhibiting cellular and spatial heterogeneity within individuals and among different patients. To gain a comprehensive understanding of the transcriptomic landscape of human knee articular cartilage, we performed extensive integration analyses using scRNA-seq and spatially resolved transcriptomic technologies for both patients with OA and non-OA controls. We identified 33 marker genes that were used for annotating 11 putative chondrocyte populations, including 8 commonly shared populations between patients with OA and non-OA controls; and 3 OA cartilage-specific populations, including 2 novel defined populations: preInfC and InfC. Our analysis revealed that chondrocytes in patients with OA exhibited high interpatient heterogeneity and represented more diverse phenotypic variations compared with controls, highlighting the need for precision medicine approaches in OA treatment.

**Figure 6:**
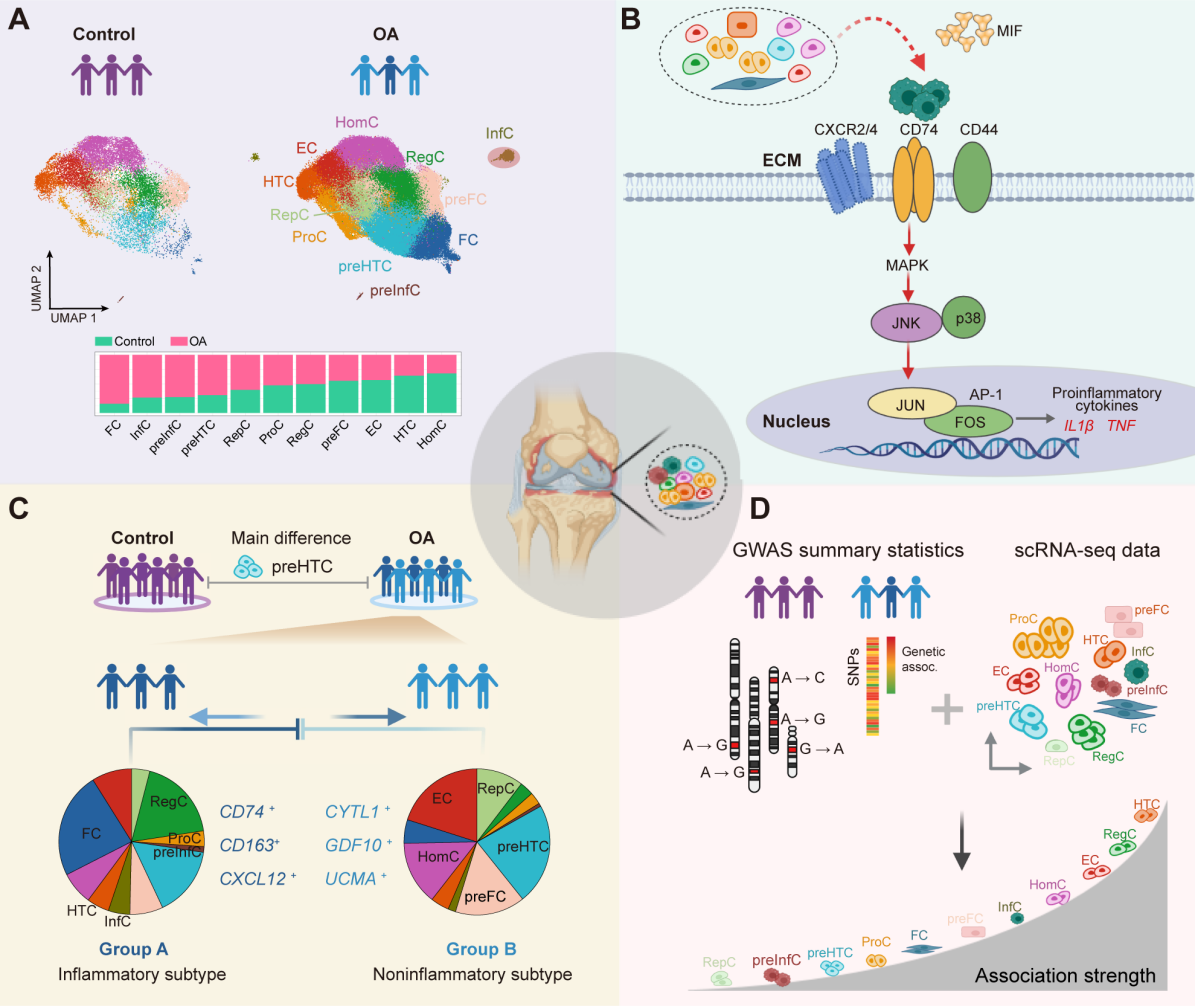
The summary of the findings. (A) The cell fraction difference between patients with OA and non-OA controls. The InfC and preInfC are newly identified chondrocyte populations, which are largely absent in non-OA controls. (B) The MIF expressed by other chondrocytes interacts with the CD74-CXCR2/4-CD44 complex expressed by InfCs and activates the MAPK signalling pathway, which increases the expression of the activator protein 1 (AP-1) transcription factor and further promotes the transcription of pro-inflammatory cytokines. This figure was created by BioRender. (C) An integrative analysis of scRNA-seq data and bulk RNA-seq data demonstrates two major subtypes of patients with OA are stratified by preFC, resulting in an inflammatory-related subtype (group A) and a non-inflammatory-related subtype of OA (group B). (D) An integrative analysis of scRNA-seq data and GWAS summary data shows that HTC is the key chondrocyte population that drives the susceptibility to the pathogenesis of OA. CXCR2/4, C-X-C motif chemokine receptor 2/4; ECM, extracellular matrix; GWAS, genome-wide association studies; HTC, hypertrophic chondrocyte; InfC, inflammatory chondrocyte; MIF, macrophage migration inhibitory factor; MAPK, mitogen-activated protein kinase; OA, osteoarthritis; preFC, prefibrocartilage chondrocyte; preInfC, pre-inflammatory chondrocyte; scRNA-seq, single-cell RNA sequencing.

While inflammatory mediators have been implicated in the regulation of cartilage damage,^9^ the role of chondrocytes in the immune response remained unclear. Recently, a scRNA-seq study for dissecting the heterogeneity of articular cartilage could detect *CD74*, *CD80*, *CD86* and *HLA-DPA1* genes that were highly expressed in a small proportion of chondrocytes, indicating that these chondrocytes may have immune cell functions.^10^ However, these chondrocytes were mixed together with the RegC population, presumably due to the limited number of chondrocytes profiling by Smart-seq2 technology^75^ (ie, 60–120 chondrocytes were profiled per sample). Another study used cytometry by time of flight (cyTOF) profiling to map the inflammatory landscape, further confirming the existence of the inflammatory populations in the articular cartilage of OA.^24^ However, it remains unclear how the cross-talk or cell communication among different chondrocyte populations happens, and the key genes or signalling pathways that might contribute to OA, presumably due to the preselected panel of 33 markers. In this study, we applied 10x Genomics scRNA-seq protocols to profile 135 896 chondrocytes, representing a comprehensive survey of the chondrocytes in OA status. Of note, we identified that the preInfC and InfC were newly expanded chondrocyte populations (a total of 1499 chondrocytes, 1.1% of all chondrocytes for InfC, and a total of 533 chondrocytes, 0.39% of all chondrocytes for preInfC) that may potentially contribute to perpetuating cartilage degradation in OA (figure 6A). The InfCs highly express *CD74*, *CXCL8* and *GPR183* genes, and were enriched for MIF-CD74 and MIF-CXCR2/4 L-R pairs, as major upstream regulator inflammatory of p38 and/or JNK MAP kinase signalling. MIF-CD74 and MIF-CXCR2/4 are essential for promoting the transcription of pro-inflammatory cytokines and complement systems (figure 6B). Therefore, targeting the MIF-CD74 or MIF-CXCR2/4 pathways is a potential therapeutic strategy for treating OA in the future.

Besides the single-cell transcriptomic landscape, we systematically characterised the transcriptomic landscape of the spatial organisation of knee articular cartilage, and revealed that the AS was the most transcriptionally active zone either in patients with OA or non-OA controls. Notably, many potential previously reported targets of candidate drugs for treating OA were detected in the AS. For example, *PRG4* known as lubricin, may contribute to smooth joint motion^76^; *CRTAC1* has been considered as a potential biomarker for early diagnosis of OA at risk for the disease earlier and monitoring the disease course.^17^ As we expected, the relatively quiescent articular chondrocytes reside on MZ and DZ of patients with OA. Overall, the integrative analysis of scRNA-seq and Geo-seq data demonstrated that most of the chondrocyte populations were located in MZ and DZ, while preHTCs and preFCs were mainly distributed in AS and SZ of patients with OA, suggesting these chondrocyte populations were potential disease-modifying candidates for treating OA.

We further showed that the preHTC contributes to OA and preFC potentially prevents cartilage degradation. We found that preHTC was significantly abundant in OA compared with non-OA controls, and preFC was abundant in the AS and SZ of WB compared with NWB. These findings were also validated by deconvolution analysis of two bulk RNA-seq data. Specifically, we found that the preHTC population was the major population to distinguish patients with OA and non-OA controls, suggesting the marker genes of this chondrocyte population are potential biomarkers for OA status. On the other hand, the preFC population is the major contributor to subtyping patients with OA (figure 6C). As a result, the preFC-depleted group was related to the inflammation-related pathways, while the preFC-abundant group was related to cartilage-related pathways. Of note, these observations need to be further validated with large-scale OA studies with comprehensive clinical features in the future.

Furthermore, we performed enrichment analysis for chondrocyte population-specific marker genes in GWAS variants in OA to investigate the OA-critical chondrocyte populations. We found that HTC was a key mediator in both European and East Asian ancestry populations. Importantly, the integrative analysis of scRNA-seq data with GWAS summary data enhances the comprehension of disease mechanisms. It also offers a more precise methodology for unravelling how genetic variants contribute to disease risk and identifying the pertinent cell types involved (figure 6D).

While vast numbers of single-cell molecular profiles have been generated, a standardised terminology for annotating cell types of knee articular cartilage tissues remains lacking. Defining cell types is challenging,^77^ probably due to factors such as unwanted variations, cellular heterogeneity, genetics, cell lineage and cell cycle phases. Recent studies have highlighted the impact of the senescent cell population (expressed: *CDKN1A*)^25 74 78^ and the stem/progenitor cell population (expressed: *CD44, ICAM1* and *VCAM1*)^79^ in the OA cartilage tissue. In particular, we also could identify these cells in ProC, but cannot expand new cell populations presumably due to the small number of chondrocytes that were assayed by scRNA-seq protocols. A new recently defined cell population MTC,^25^ which we could also identify in a subpopulation of preHTC—preHTC-1, which highly expressed *MT1M*, *MT1A* and *MT1G* (figure 4C). In summary, we provide a spatially aware annotation of cell populations in human knee articular cartilage and perform independent validation using publicly available scRNA-seq data. However, a holistic view of cell populations in human knee articular cartilage remains elusive. We emphasise the importance of comprehensive screening and experimental validation to investigate marker gene candidates that are generally applicable for annotating cell populations.

Acknowledging the limitation of small sample size in non-OA control spatial data, we recognise the potential bias in identifying non-representative DE genes. Despite this constraint, we increased the assayed spots in each zone and conducted additional validations to ensure the biologically meaningfulness of these DE genes. Together, our study presents a transcriptional landscape of chondrocytes in knee articular cartilage at both single-cell and zone-level spatial resolutions in patients with OA and patients without OA. The new findings have the potential to enhance diagnosis and treatment, provide insights into key molecular players in OA and uncover promising drugs for personalised medicine strategies in OA.

## Materials and methods

### Cartilage sample collection

We recruited eight patients with OA who met the Modified Outerbridge Classification criteria and underwent knee arthroplasty surgery, and obtained the articular cartilage tissue. We also recruited three normal controls who had undergone amputation and had no history of joint injury or disease. The knee cartilage samples were obtained from the discarded tissue after knee arthroplasty. Subjects were excluded if they had or had previously suffered from any other osteoarticular disease (eg, rheumatoid arthritis, gout or skeletal fluorosis), any other type of macrosomia, a disorder of osteochondrodysplasic or chronic disease (eg, hypertension, diabetes or coronary heart disease) or had received any treatment in the previous 6 months. The basic physiological characteristics (six females and six males; 64.33±7.6 years for patients with OA and 51.67±4.9 years for non-OA controls) of the recruited samples are shown in online supplemental table S1. In addition, six patients with OA and six non-OA controls were used for immunohistochemical validation (online supplemental table S15).

### Chondrocyte isolation and library construction for 10x Genomics protocol

Cartilage was harvested within an hour after surgery, washed twice with phosphate-buffered saline (PBS) containing penicillin and streptomycin, and cut into 1 mm^3^ pieces. Samples were then subjected to pretreatment with 0.25% trypsin (Thermo Fisher Scientific, Waltham, Massachusetts, USA) at 37°C in an incubator with 5% CO_2_ for 30 min. The cartilage fragment was then centrifuged at 350×g for 5 min and the supernatant was discarded completely, followed by digestion in basal Dulbecco's Modified Eagle Medium/F12 media (Gibico, Grand Island, New York, USA) supplemented with 0.2% type II collagenase (Gibico) at 37°C for 10–12 hours. Cell count and viability were estimated using a fluorescence Cell Analyzer (Countstar Rigel S2) with Acidine Orange/Propidium Iodide (AO/PI) reagent after the removal of erythrocytes (Miltenyi 130-094-183). Debris and dead cells removal was performed according to the recommended manuals (Miltenyi 130-109-398/130-090-101). For the 10x Genomics protocol, isolated chondrocytes were filtered through a 70 μm cell strainer, washed twice with PBS buffer and directly subjected to complementary DNA (cDNA) library construction.^10 80^ Articular cartilage tissue was dissociated into single cells by enzymatic methods according to standard procedures. Briefly, the cells were washed with PBS and resuspended in 500 μL PBS, and single-cell RNA-seq libraries were prepared using the Chromium Single Cell 3’ Library V.3 Reagent Kit (10x Genomics, Pleasanton, California, USA). Sequencing was performed by Illumina NovaSeq 6000.

### Processing of scRNA-seq data

Unique molecular identifier (UMI) counts were obtained by aligning FASTQ files to the human reference genome (GRCh38 3.0.0) through Cell Ranger (V.4.0) software from the 10x Genomics website (https://support.10xgenomics.com/single-cell-gene-expression/software/pipelines/latest/advanced/references). Gene expression raw count matrices were used for downstream analyses. Specifically, for each scRNA-seq dataset, the quality control steps included low-quality cell removal and mitochondrial count filtering. The quality control for each scRNA-seq dataset is summarised in online supplemental table S2. We performed the doublets removal step using the *DoubletFinder* (V.2.0.3)^81^ when the captured cell was >10 000, with pK (the principal component neighbourhood size used to calculate pANN) set to 0.09, nEXP (the total number of doublet predictions produced) set to 0.075 and other parameters as the default settings.

### Geo-sequencing

Fresh cartilage tissue obtained from patients with OA and non-OA controls was placed in cold RNase-free PBS. The tissue was then gently transferred to precooled optimum cutting temperature (OCT) compound, ensuring that the entire tissue was embedded. The tissue was immediately frozen in liquid nitrogen or dry ice and transferred to −80°C. The OCT-embedded samples were cryosectioned at −20°C at 14 μm thickness. The sections were then mounted on polyethylene-terephthalate-coated slides, fixed immediately with ethanol and stained with 1% cresyl violet acetate dissolved in 75% ethanol solution. Afterwards, targeted regions of sections were collected by LCM, and transcriptome analyses of those collected samples were performed by Geo-seq.^36^ cDNA library was prepared using TruePrep DNA Library Prep Kit V2 for Illumina (Vazyme #TD501-503) and sequenced on Illumina NovaSeq 6000 with PE150 mode. We classified the zones for knee articular cartilage based on the organisation and degree of alignment of the collagen fibers, which could be summarised as the following criteria (online supplemental figure S4A): (1) the hyaline superficial cartilage of the articular cartilage was recognised as the AS; (2) the thin SZ of the remaining cartilage, where the collagen fibers are densely packed and aligned parallel to the AS, and the chondrocytes are flattened, was recognised as the SZ; (3) the zone where the collagen fibers are obliquely organised and the chondrocytes are spherical and of low density was recognised as the MZ; (4) the zone where the collagen fibers are arranged perpendicular to the AS and the chondrocytes are hypertrophic was recognised as the DZ. To avoid overlap between zones, regions of approximately 200 μm between SZ and MZ and MZ and DZ were discarded.

### Processing of Geo-seq data

We performed an accurate alignment method with the reference genomes (hg19, http://genome.ucsc.edu/index.html) using HISAT2 software^82^ (https://github.com/DaehwanKimLab/hisat2, V.2.2.1) to convert FASTQ files into BAM files. We then performed the *featureCounts* 83 method with basic gene annotation (ftp://ftp.ebi.ac.uk/pub/databases/gencode/Gencode_human/release_26/GRCh37_mapping/gencode.v26lift37.annotation.gtf.gz) to quantify RNA-seq gene expression levels. Gene expression raw count matrices were used for downstream analyses.

### Integrative analysis of scRNA-seq datasets

To assess the reproducibility of chondrocyte annotations across 19 scRNA-seq datasets from 11 individuals, we performed an in-depth integrative analysis. We performed integrative analyses using Harmony,^84^ LIGER^85^ and Seurat CCA^86^ to provide independent validations of chondrocyte annotations. In particular, we observed a similar number and composition of chondrocyte populations in all scenarios, regardless of the number of latent dimensions (online supplemental figures S15A-S15C) or the choice of integrative analysis methods (online supplemental figures S15C and S15D). To remove dataset-specific batch effects, we evaluated all integrated results according to an existing benchmark pipeline.^87^ We compared different integration methods (ie, Harmony, LIGER and Seurat) with a range of number of PCs and clustering resolutions. In the first step, the robustness of the clustering was tested by comparing the results under different resolutions, resolutions that guaranteed all chondrocyte populations were detected as separate clusters were selected. In the second step, different integration methods were benchmarked through a pipeline according to a previous study.^87^ Finally, the optimal integration methods (ie, Harmony) were selected for the downstream analysis with PCs=30 and resolution=0.9, to produce an integrated embedding of all chondrocytes (online supplemental figure S15A).

Specifically, we performed integrative analyses following the Harmony pipeline^84^ (https://htmlpreview.github.io/?https://github.com/immunogenomics/harmony/blob/master/doc/quickstart.html), the Seurat pipeline^22 88^ (https://satijalab.org/seurat/articles/integration_introduction.html) and the LIGER pipeline (http://htmlpreview.github.io/?https://github.com/welch-lab/liger/blob/master/vignettes/Integrating_multi_scRNA_data.html). For the Harmony pipeline (V.1.0.3), scRNA-seq data from different samples were merged into one Seurat object, then normalised using the *NormalizeData* function and scaled on 2000 most variable features using the *FindVariableFeatures* and *ScaleData* functions. Principal component analysis (PCA) was performed using the *RunPCA* function, followed by the *RunHarmony* function to correct for batch effects across samples. For the Seurat pipeline, we followed the routine integration pipeline (V.4.2.0), that is, the log-normalisation was performed using the *NormalizeData* function; selection of highly variable genes was performed using the *FindVariableFeatures* function (VST method); correction of batch effects across samples was performed using the *FindIntegrationAnchors* and *IntegrateData* functions; dimensionality reduction was performed using the *RunPCA* and *RunUMAP* functions; neighbour network construction was performed using the *FindNeighbors* function. For the LIGER pipeline, the log-normalisation was performed using the normalise function; selection of highly variable genes was performed using the *selectGenes* function; batch effects correction across samples was performed using the *optimizeALS* and *quantile_norm* functions; clustering was performed using *louvainCluster* function, and the marker genes for each population were identified using the *FindMarkers* function with the Wilcoxon rank-sum test for all pipelines.

### Cell compositional analysis

We ran Cacao (V.0.4.0)^59^ to assess the changes in cell composition between patients with OA and non-OA controls for each chondrocyte population. The statistical significance of the difference in cell composition was tested using the *estimateCellLoadings* function. To test the statistical significance of cell composition changes in the deconvolution results of Geo-seq data and bulk RNA-seq data, we performed a permutation t-test implemented in the exactRankTests R package (V.0.8.35).

### Trajectory inference analysis

We ran monocle2 (V.2.18.0)^89^ on the raw counts of the scRNA-seq data. After calculating size factors and estimating dispersions using the *estimateSizeFactors* and *estimateDispersions* functions, we identified 13 124 DE genes along the lineage using a trajectory-based differential gene test. We selected the top 3000 genes ranked by q-value generated by the *differentialGeneTest* function. Dimensionality reduction analysis was performed using the DDRTree method. After cell ordering, the trajectory was visualised using the *plot_cell_trajectory* function with default parameter settings.

### Cell-cell communication analysis

Cell-cell interaction analyses were performed on the patient with OA and the non-OA control, using CellChat (V.1.4.0).^50^ Specifically, CellChat was run on each group, and the log-normalised expression data and cell annotation information were used for analysis. We followed the standard pipeline (https://github.com/sqjin/CellChat/blob/master/tutorial/CellChat-vignette.html) to perform all analyses using the default parameters and the human ligand-receptors database.

### Deconvolution analysis

We performed the deconvolution analysis using CIBERSORTx (https://cibersortx.stanford.edu/)^46^ for two different scenarios: (1) integration of the scRNA-seq data with Geo-seq data to determine the chondrocyte composition on the spatial landscape of cartilage tissue and (2) integration the scRNA-seq data with traditional bulk RNA-seq data to decipher the chondrocyte composition of patients with OA. Specifically, we first constructed the gene signature matrix using the top marker genes (log2FC>0.25 and adjusted p<0.05) for all chondrocyte populations. The deconvolution analysis was performed in S-mode due to the possibility of high technical variance. All samples were generated with the p values that were included in the downstream analysis if the p value was <0.05. Finally, a permutation test^59^ was performed to test whether or not the cell proportions were significantly different between patients with OA and non-OA controls or not.

### Differential expression analysis between patients with OA and non-OA controls

We identified DE genes between patients with OA and non-OA controls for each common cell population. Briefly, we first aggregated UMI counts within a given chondrocyte population to generate pseudobulk RNA-seq data. We then performed DESeq2^47^ implemented in Cacoa^59^ to identify DE genes using a likelihood ratio test. We considered a gene with |log2FC|>1 and adjusted p<0.05 (the multiple testing correction is the Benjamini and Hochberg) as a DE gene. We excluded InfC, preInfC and FC when performing DE analysis between patients with OA and non-OA controls because they were absent in non-OA samples. We also performed DESeq2 to identify DE genes between patients with OA and non-OA controls for Geo-seq data.

### Gene set enrichment analysis

Gene set enrichment analyses were conducted using the *enrichGO* function implemented in the clusterProfiler R package (V.3.18.1).^90^ Specifically, the genes retained after QC were used as background genes, and the minimum and maximum sizes of genes annotated with GO terms for testing were set to 10 and 500, respectively. The significant GO terms were selected with adjusted p<0.05 (the multiple testing correction is the Benjamini and Hochberg).

### Subpopulation analysis of chondrocytes

We performed the subclustering analysis of InfC, preInfC and preHTC populations using *FindSubCluster* function that was implemented in Seurat.^22 88^ Specifically, we performed an integrative analysis of InfCs, preInfCs from 16 OA samples, performed an integrative analysis of preHTCs from all 19 samples and then applied dimensionality reduction (*RunPCA*, *k*=30) and clustering (*FindCluster*, Louvain algorithm, *resolution*=0.1) to determine the preInfC, InfC and preHTC subpopulations.

### Defining inflammatory score of chondrocytes

The inflammatory score was used to evaluate the inflammatory degree of individual chondrocytes expressed in a certain predefined expression gene set obtained from a previous study.^58^ Such scores were calculated using the *AddModuleScore* function in Seurat, with default parameter settings. We used the median of the inflammatory score of the given chondrocyte subpopulation as the representative score.

### Identifying OA status-associated chondrocytes

To investigate OA-associated chondrocytes, we performed the Scissor analysis (V.2.0.0)^66^ on the scRNA-seq data and two bulk RNA-seq datasets (ie, GSE114007 and E-MTAB-4304), and set the L1 norm and network-based penalties balance parameter α=0.02. The chondrocytes that negatively and positively correlated with the phenotype were labelled as Scissor^−^ and Scissor^+^, respectively.

### Identifying OA-associated subnetworks

We performed an OA-associated sub-network analysis using PhenomeExpress.^91^ The human ConsensusPathDB (V.0.0.30) network^92^ was experimentally derived from protein-protein interactions. Gene symbol ID conversion was performed using BioMart (V.2.46.3). Phenotypes relevant to OA were selected from the UberPheno ontology (MP: 0003436, HP: 0001387, MP: 0003724, HP: 0002758).

### Classification using logistic regression models

We used the logistic regression model using the *LinearRegression* function, which was implemented in *sklearn* (https://scikitlearn.org/stable/) to predict OA status. The parameter settings were *penalty*=*‘elasticnet’* and *solver*=*‘saga’* for prediction model training. The discriminative genes were selected as DE genes of each chondrocyte population using pseudobulk analysis. LOOCV was used for model training. The performance of the classification models was assessed by the AUC.

### The validation of DE genes in PubMed

We used the RISmed R package (V.2.3.0) to validate the detected DE genes and enriched pathways between patients with OA and non-OA controls in Geo-seq data. First, we searched PubMed with the term ‘osteoarthritis, cartilage, chondrocyte’ and the gene symbol of all analysed genes from 1 January 2000 to 12 December 2023 selecting for primary research studies. we used the function *EUtilsQuery* to extract bibliographic content from PubMed. Out of the 14 062 genes, a total of 4710 had at least one publication. We considered those genes with ≥1 publication (the 50% quantile of the number of publications) as OA-associated genes, resulting in a total of 2886 genes.

We defined OA-related pathways with a similar approach. Out of the 22 874 pathways obtained from the MSigDB C5 collection, 12 990 had at least one publication. We considered pathways with ≥12 publications (the 50% quantile of the number of publications) as OA-associated pathways, resulting in a total of 6552 pathways.

### Immunohistochemistry staining

Knee cartilage tissue sections were deparaffined in xylene and hydrated in gradient ethanol. After three times PBS washing, sections were incubated with 3% H_2_O_2_ for 15 min to block endogenous catalase. Then tissue sections were incubated with prewarmed 1% trypsin at 37°C for 30 min for antigen retrieval. After rinsing, sections were sealed with goat serum (ZSGB-BIO, China) for 20 min at room temperature and incubated with rabbit antihuman CD74 (1:50, ab181470, Abcam, UK), mouse antihuman BMP2 (1:100, 66383-1-Ig, Proteintech, China), rabbit antihuman IBSP (1:2000, ab270605, Abcam), rabbit antihuman CHI3L1 (1:200, ab255297, Abcam), rabbit antihuman GPR183 (1:200, ab150625, Abcam), rabbit antihuman BAG1 (1:100, 19064-1-AP, Proteintech), rrabbit antihuman CD9 (1:2000, 20597-1-AP, Proteintech) overnight at 4°C. Then tissue sections were incubated with goat antimouse secondary antibody (ZSGB-BIO, China) for 30 min at 37°C. After staining with 3,3'-diaminobenzidine and counterstaining with haematoxylin, tissue sections were dehydrated in gradient ethanol and sealed with neutral balsam mounting medium. Typical positive cells were captured under a microscope at 400× magnification.

### Lentivirus construction and amplification

CD74 overexpressing lentivirus was directly synthesised and amplified by Tsingke (China). In brief, the coding sequence of CD74 was cloned into lentiviral vector PDS279_pL-CMV-GFP-ccdB-puro (Tsingke, China). The virus particle was constructed and amplified in HEK293T cells and collected by ultracentrifugation. The supernatant containing the lentiviral particles was collected at 48 and 60 hours after transfection and mixed before ultracentrifugation at 19 400g for 2.5 hours at 4°C. We seeded human chondrocyte cell line C28/I2 on the 12-well plate and infected with CD74 overexpressing lentivirus and control lentivirus at multiplicity of infection 100. Cells were harvested at 72 hours after infection for subsequent analysis. The results of qRT-PCR were plotted using GraphPad PRISM V.6 (GraphPad, San Diego, California, USA).

### Quantitative real-time PCR

Cells were lysed and total RNA was extracted using a commercial kit (Omega, Connecticut, USA). Total RNA was then reversely transcribed using PrimeScript RT Master Mix (Takara, Japan) and cDNA was quantified using TB Green Premix (Takara). For each gene, the relative mRNA expression level was normalised to the expression level of β-actin and calculated using the 2^−∆∆CT^ method. The primers used in this study are listed in online supplemental table S16. The results of qRT-PCR were presented in bar plots using GraphPad PRISM V.6 (GraphPad).

### GWAS summary data from the UK Biobank

The GWAS data for this paper were obtained from the UK Biobank under an approved application (ID: 67665). We stratified the participants who are from East Asia and self-reported experiencing knee pain (Data-Field 6159, n=1172 individuals, accessed 20 June 2022). Controls were defined as participants who did not suffer any pain (n=807 individuals, accessed 20 June 2022). The 1000 Genomes Project haplotype reference panel^93^ (https://www.internationalgenome.org) was used to impute untyped variants via the Michigan imputation server. After genotype imputation and quality control (single nucleotide polymorphisms with minor allele frequency >1%, and r^2^ >0.99 when imputed), the number of variants was 1 030 454. We performed genome-wide association analysis using GMMAT,^71^ including age and sex as covariates. GWAS summary statistics for European ancestry were obtained using linear regression models from the Neale’s laboratory (http://www.nealelab.is/uk-biobank).

### Patient and public involvement

Patients and/or the public were not involved in the design, conduct, reporting, or dissemination plans of this research.

## Data Availability

Data may be obtained from a third party and are not publicly available. Single-cell RNA-seq data are available at the NCBI's Gene Expression Omnibus (GEO) data repository with the accession ID GSE255460 and Geo-seq data are available at the NCBI's GEO data repository with the accession ID GSE254844.

## References

[1] Pigeolet M , Jayaram A , Park KB , et al . Osteoarthritis in 2020 and beyond. Lancet 2021;397:1059–60. 10.1016/S0140-6736(21)00208-7 33743863

[2] Collaborators . Global burden of 369 diseases and injuries in 204 countries and territories, 1990–2019: a systematic analysis for the Global Burden of Disease Study 2019. The Lancet 2020;396:1204–22. 10.1016/S0140-6736(20)30925-9 PMC7567026 33069326

[3] Chen D , Shen J , Zhao W , et al . Osteoarthritis: toward a comprehensive understanding of pathological mechanism. Bone Res 2017;5:16044. 10.1038/boneres.2016.44 28149655 PMC5240031

[4] Bernabei I , So A , Busso N , et al . Cartilage calcification in osteoarthritis: mechanisms and clinical relevance. Nat Rev Rheumatol 2023;19:10–27. 10.1038/s41584-022-00875-4 36509917

[5] Hügle T , Geurts J . What drives osteoarthritis?-synovial versus subchondral bone pathology. Rheumatology (Oxford) 2017;56:1461–71. 10.1093/rheumatology/kew389 28003493

[6] Lee W , Nims RJ , Savadipour A , et al . Inflammatory signaling sensitizes Piezo1 mechanotransduction in articular chondrocytes as a pathogenic feed-forward mechanism in osteoarthritis. Proc Natl Acad Sci USA 2021;118:e2001611118. 10.1073/pnas.2001611118 33758095 PMC8020656

[7] Berenbaum F . Osteoarthritis as an inflammatory disease (osteoarthritis is not osteoarthrosis!). Osteoarthritis and Cartilage 2013;21:16–21. 10.1016/j.joca.2012.11.012 23194896

[8] Goldring MB , Otero M , Tsuchimochi K , et al . Defining the roles of inflammatory and anabolic cytokines in cartilage metabolism. Ann Rheum Dis 2008;67 Suppl 3:iii75–82. 10.1136/ard.2008.098764 19022820 PMC3939701

[9] van den Bosch MHJ . Inflammation in osteoarthritis: is it time to dampen the alarm(in) in this debilitating disease? Clin Exp Immunol 2019;195:153–66. 10.1111/cei.13237 30421798 PMC6330652

[10] Ji Q , Zheng Y , Zhang G , et al . Single-cell RNA-seq analysis reveals the progression of human osteoarthritis. Ann Rheum Dis 2019;78:100–10. 10.1136/annrheumdis-2017-212863 30026257 PMC6317448

[11] Soul J , Dunn SL , Anand S , et al . Stratification of knee osteoarthritis: two major patient subgroups identified by genome-wide expression analysis of articular cartilage. Ann Rheum Dis 2018;77:423–30. 10.1136/annrheumdis-2017-212603 29273645 PMC5867416

[12] Ratneswaran A , Rockel JS , Kapoor M . Understanding osteoarthritis pathogenesis: a multiomics system-based approach. Curr Opin Rheumatol 2020;32:80–91. 10.1097/BOR.0000000000000680 31724972

[13] McDonald M-LN , Lakshman Kumar P , Srinivasasainagendra V , et al . Novel genetic loci associated with osteoarthritis in multi-ancestry analyses in the Million Veteran Program and UK Biobank. Nat Genet 2022;54:1816–26. 10.1038/s41588-022-01221-w 36411363

[14] Tachmazidou I , Hatzikotoulas K , Southam L , et al . Identification of new therapeutic targets for osteoarthritis through genome-wide analyses of UK Biobank data. Nat Genet 2019;51:230–6. 10.1038/s41588-018-0327-1 30664745 PMC6400267

[15] Yuan C , Pan Z , Zhao K , et al . Classification of four distinct osteoarthritis subtypes with a knee joint tissue transcriptome atlas. Bone Res 2020;8:38. 10.1038/s41413-020-00109-x 33298863 PMC7658991

[16] Fan Y , Gao D , Zhang Y , et al . Genome-Wide Differentially Methylated Region Analysis to Reveal Epigenetic Differences of Articular Cartilage in Kashin–Beck Disease and Osteoarthritis. Front Cell Dev Biol 2021;9:636291. 10.3389/fcell.2021.636291 33732704 PMC7957013

[17] Styrkarsdottir U , Lund SH , Saevarsdottir S , et al . The CRTAC1 Protein in Plasma Is Associated With Osteoarthritis and Predicts Progression to Joint Replacement: A Large-Scale Proteomics Scan in Iceland. Arthritis Rheumatol 2021;73:2025–34. 10.1002/art.41793 33982893 PMC8596997

[18] Camacho-Encina M , Balboa-Barreiro V , Rego-Perez I , et al . Discovery of an autoantibody signature for the early diagnosis of knee osteoarthritis: data from the Osteoarthritis Initiative. Ann Rheum Dis 2019;78:1699–705. 10.1136/annrheumdis-2019-215325 31471297 PMC6900252

[19] Stark R , Grzelak M , Hadfield J . RNA sequencing: the teenage years. Nat Rev Genet 2019;20:631–56. 10.1038/s41576-019-0150-2 31341269

[20] Goodwin S , McPherson JD , McCombie WR . Coming of age: ten years of next-generation sequencing technologies. Nat Rev Genet 2016;17:333–51. 10.1038/nrg.2016.49 27184599 PMC10373632

[21] Lee J , Hyeon DY , Hwang D . Single-cell multiomics: technologies and data analysis methods. Exp Mol Med 2020;52:1428–42. 10.1038/s12276-020-0420-2 32929225 PMC8080692

[22] Stuart T , Satija R . Integrative single-cell analysis. Nat Rev Genet 2019;20:257–72. 10.1038/s41576-019-0093-7 30696980

[23] Marx V . Method of the Year: spatially resolved transcriptomics. Nat Methods 2021;18:9–14. 10.1038/s41592-020-01033-y 33408395

[24] Grandi FC , Baskar R , Smeriglio P , et al . Single-cell mass cytometry reveals cross-talk between inflammation-dampening and inflammation-amplifying cells in osteoarthritic cartilage. Sci Adv 2020;6:eaay5352. 10.1126/sciadv.aay5352 32201724 PMC7069698

[25] Swahn H , Li K , Duffy T , et al . Senescent cell population with ZEB1 transcription factor as its main regulator promotes osteoarthritis in cartilage and meniscus. Ann Rheum Dis 2023;82:403–15. 10.1136/ard-2022-223227 36564153 PMC10076001

[26] Martel-Pelletier J , Barr AJ , Cicuttini FM , et al . Osteoarthritis. Nat Rev Dis Primers 2016;2:16072. 10.1038/nrdp.2016.72 27734845

[27] Sophia Fox AJ , Bedi A , Rodeo SA . The basic science of articular cartilage: structure, composition, and function. Sports Health 2009;1:461–8. 10.1177/1941738109350438 23015907 PMC3445147

[28] Liu C-F , Samsa WE , Zhou G , et al . Transcriptional control of chondrocyte specification and differentiation. Seminars in Cell & Developmental Biology 2017;62:34–49. 10.1016/j.semcdb.2016.10.004 27771362 PMC5318237

[29] Loeser RF . Aging and osteoarthritis: the role of chondrocyte senescence and aging changes in the cartilage matrix. Osteoarthritis and Cartilage 2009;17:971–9. 10.1016/j.joca.2009.03.002 19303469 PMC2713363

[30] Musumeci G , Castrogiovanni P , Trovato F , et al . Biomarkers of Chondrocyte Apoptosis and Autophagy in Osteoarthritis. IJMS 2015;16:20560–75. 10.3390/ijms160920560 26334269 PMC4613218

[31] Héraud F , Héraud A , Harmand MF . Apoptosis in normal and osteoarthritic human articular cartilage. Ann Rheum Dis 2000;59:959–65. 10.1136/ard.59.12.959 11087699 PMC1753049

[32] Styrkarsdottir U , Lund SH , Thorleifsson G , et al . Meta-analysis of Icelandic and UK data sets identifies missense variants in SMO, IL11, COL11A1 and 13 more new loci associated with osteoarthritis. Nat Genet 2018;50:1681–7. 10.1038/s41588-018-0247-0 30374069

[33] Zengini E , Hatzikotoulas K , Tachmazidou I , et al . Genome-wide analyses using UK Biobank data provide insights into the genetic architecture of osteoarthritis. Nat Genet 2018;50:549–58. 10.1038/s41588-018-0079-y 29559693 PMC5896734

[34] Boer CG , Hatzikotoulas K , Southam L , et al . Deciphering osteoarthritis genetics across 826,690 individuals from 9 populations. Cell 2021;184:4784–4818. 10.1016/j.cell.2021.07.038 34450027 PMC8459317

[35] Zheng GXY , Terry JM , Belgrader P , et al . Massively parallel digital transcriptional profiling of single cells. Nat Commun 2017;8:14049. 10.1038/ncomms14049 28091601 PMC5241818

[36] Chen J , Suo S , Tam PP , et al . Spatial transcriptomic analysis of cryosectioned tissue samples with Geo-seq. Nat Protoc 2017;12:566–80. 10.1038/nprot.2017.003 28207000

[37] Blaney Davidson EN , Vitters EL , Mooren FM , et al . Connective tissue growth factor/CCN2 overexpression in mouse synovial lining results in transient fibrosis and cartilage damage. Arthritis Rheum 2006;54:1653–61. 10.1002/art.21795 16646035

[38] Shen J , Abu-Amer Y , O’Keefe RJ , et al . Inflammation and epigenetic regulation in osteoarthritis. Connect Tissue Res 2017;58:49–63. 10.1080/03008207.2016.1208655 27389927 PMC5266560

[39] Sandell LJ . Anabolic factors in degenerative joint disease. Curr Drug Targets 2007;8:359–65. 10.2174/138945007779940142 17305513

[40] Qin X , Jiang Q , Nagano K , et al . Runx2 is essential for the transdifferentiation of chondrocytes into osteoblasts. PLoS Genet 2020;16:e1009169. 10.1371/journal.pgen.1009169 33253203 PMC7728394

[41] Jiang L , Zheng Z , Fang H , et al . A generalized linear mixed model association tool for biobank-scale data. Nat Genet 2021;53:1616–21. 10.1038/s41588-021-00954-4 34737426

[42] Mokuda S , Nakamichi R , Matsuzaki T , et al . Wwp2 maintains cartilage homeostasis through regulation of Adamts5. Nat Commun 2019;10:2429. 10.1038/s41467-019-10177-1 31160553 PMC6546747

[43] Fan J-Q , Miao Y-T , Lu K-C , et al . A IFI27 gene contributes to ER-stress mediated apoptosis and benefits for white spot syndrome virus infection in Litopenaeus vannamei. Fish & Shellfish Immunology 2022;120:180–9. 10.1016/j.fsi.2021.11.032 34838985

[44] Ashraf S , Walsh DA . Angiogenesis in osteoarthritis. Curr Opin Rheumatol 2008;20:573–80. 10.1097/BOR.0b013e3283103d12 18698180

[45] Mapp PI , Walsh DA . Mechanisms and targets of angiogenesis and nerve growth in osteoarthritis. Nat Rev Rheumatol 2012;8:390–8. 10.1038/nrrheum.2012.80 22641138

[46] Newman AM , Steen CB , Liu CL , et al . Determining cell type abundance and expression from bulk tissues with digital cytometry. Nat Biotechnol 2019;37:773–82. 10.1038/s41587-019-0114-2 31061481 PMC6610714

[47] Love MI , Huber W , Anders S . Moderated estimation of fold change and dispersion for RNA-seq data with DESeq2. Genome Biol 2014;15:550. 10.1186/s13059-014-0550-8 25516281 PMC4302049

[48] Grogan SP , Duffy SF , Pauli C , et al . Zone-specific gene expression patterns in articular cartilage. Arthritis Rheum 2013;65:418–28. 10.1002/art.37760 23124445 PMC3558601

[49] Su X , Shi Y , Zou X , et al . Single-cell RNA-Seq analysis reveals dynamic trajectories during mouse liver development. BMC Genomics 2017;18:946. 10.1186/s12864-017-4342-x 29202695 PMC5715535

[50] Jin S , Guerrero-Juarez CF , Zhang L , et al . Inference and analysis of cell-cell communication using CellChat. Nat Commun 2021;12:1088. 10.1038/s41467-021-21246-9 33597522 PMC7889871

[51] Calandra T , Roger T . Macrophage migration inhibitory factor: a regulator of innate immunity. Nat Rev Immunol 2003;3:791–800. 10.1038/nri1200 14502271 PMC7097468

[52] Rowe MA , Harper LR , McNulty MA , et al . Reduced Osteoarthritis Severity in Aged Mice With Deletion of Macrophage Migration Inhibitory Factor. Arthritis Rheumatol 2017;69:352–61. 10.1002/art.39844 27564840 PMC5274570

[53] Chatterjee M , Borst O , Walker B , et al . Macrophage migration inhibitory factor limits activation-induced apoptosis of platelets via CXCR7-dependent Akt signaling. Circ Res 2014;115:939–49. 10.1161/CIRCRESAHA.115.305171 25266363

[54] Kita K , Kimura T , Nakamura N , et al . PI3K/Akt signaling as a key regulatory pathway for chondrocyte terminal differentiation. Genes Cells 2008;13:839–50. 10.1111/j.1365-2443.2008.01209.x 18782222

[55] Kapoor M , Martel-Pelletier J , Lajeunesse D , et al . Role of proinflammatory cytokines in the pathophysiology of osteoarthritis. Nat Rev Rheumatol 2011;7:33–42. 10.1038/nrrheum.2010.196 21119608

[56] Molnar V , Matišić V , Kodvanj I , et al . Cytokines and Chemokines Involved in Osteoarthritis Pathogenesis. Int J Mol Sci 2021;22:9208. 10.3390/ijms22179208 34502117 PMC8431625

[57] Lu JP , Wu ZX , Xiong Y . Knockdown of long noncoding RNA HOTAIR inhibits osteoarthritis chondrocyte injury by miR-107/CXCL12 axis. J Orthop Surg Res 2021;16. 10.1186/s13018-021-02547-7 PMC8237457 34183035

[58] Wauters E , Van Mol P , Garg AD , et al . Discriminating mild from critical COVID-19 by innate and adaptive immune single-cell profiling of bronchoalveolar lavages. Cell Res 2021;31:272–90. 10.1038/s41422-020-00455-9 33473155 PMC8027624

[59] Petukhov V , Igolkina A , Rydbirk R , et al . Case-control analysis of single-cell RNA-seq studies. Bioinformatics [Preprint] 2022. 10.1101/2022.03.15.484475

[60] Squair JW , Gautier M , Kathe C , et al . Confronting false discoveries in single-cell differential expression. Nat Commun 2021;12:5692. 10.1038/s41467-021-25960-2 34584091 PMC8479118

[61] Wei L , Sun X , Wang Z , et al . CD95-induced osteoarthritic chondrocyte apoptosis and necrosis: dependency on p38 mitogen-activated protein kinase. Arthritis Res Ther 2006;8:R37. 10.1186/ar1891 16469115 PMC1526592

[62] Dunn SL , Soul J , Anand S , et al . Gene expression changes in damaged osteoarthritic cartilage identify a signature of non-chondrogenic and mechanical responses. Osteoarthritis and Cartilage 2016;24:1431–40. 10.1016/j.joca.2016.03.007 26973327 PMC4989048

[63] Aigner T , Fundel K , Saas J , et al . Large-scale gene expression profiling reveals major pathogenetic pathways of cartilage degeneration in osteoarthritis. Arthritis Rheum 2006;54:3533–44. 10.1002/art.22174 17075858

[64] Xu W , Gu S , Zhang G , et al . APOD acts on fibroblast-like synoviocyte and chondrocyte to alleviate the process of osteoarthritis in vitro. J Orthop Res 2024;42:296–305. 10.1002/jor.25690 37728985

[65] Blanco FJ , Valdes AM , Rego-Pérez I . Mitochondrial DNA variation and the pathogenesis of osteoarthritis phenotypes. Nat Rev Rheumatol 2018;14:327–40. 10.1038/s41584-018-0001-0 29670212

[66] Sun D , Guan X , Moran AE , et al . Identifying phenotype-associated subpopulations by integrating bulk and single-cell sequencing data. Nat Biotechnol 2022;40:527–38. 10.1038/s41587-021-01091-3 34764492 PMC9010342

[67] Rocha FAC , Ali SA . Soluble biomarkers in osteoarthritis in 2022: year in review. Osteoarthritis and Cartilage 2023;31:167–76. 10.1016/j.joca.2022.09.005 36179981

[68] Coutinho de Almeida R , Mahfouz A , Mei H , et al . Identification and characterization of two consistent osteoarthritis subtypes by transcriptome and clinical data integration. Rheumatology 2021;60:1166–75. 10.1093/rheumatology/keaa391 32885253 PMC7937023

[69] Neumann G , Hunter D , Nevitt M , et al . Location specific radiographic joint space width for osteoarthritis progression. Osteoarthritis and Cartilage 2009;17:761–5. 10.1016/j.joca.2008.11.001 19073368 PMC3138121

[70] Delibaltov DL , Gaur U , Kim J , et al . CellECT: cell evolution capturing tool. BMC Bioinformatics 2016;17:88. 10.1186/s12859-016-0927-7 26887436 PMC4756481

[71] Chen H , Wang C , Conomos MP , et al . Control for Population Structure and Relatedness for Binary Traits in Genetic Association Studies via Logistic Mixed Models. Am J Hum Genet 2016;98:653–66. 10.1016/j.ajhg.2016.02.012 27018471 PMC4833218

[72] Bycroft C , Freeman C , Petkova D , et al . The UK Biobank resource with deep phenotyping and genomic data. Nature 2018;562:203–9. 10.1038/s41586-018-0579-z 30305743 PMC6786975

[73] Martin A , Finucane H , Daly M , et al . GWAS round 2. 2018. Available: http://www.nealelab.is/uk-biobank/

[74] Liu Y , Zhang Z , Li T , et al . Senescence in osteoarthritis: from mechanism to potential treatment. Arthritis Res Ther 2022;24:174. 10.1186/s13075-022-02859-x 35869508 PMC9306208

[75] Picelli S , Faridani OR , Björklund AK , et al . Full-length RNA-seq from single cells using Smart-seq2. Nat Protoc 2014;9:171–81. 10.1038/nprot.2014.006 24385147

[76] Saito T . The superficial zone of articular cartilage. Inflamm Regener 2022;42:14. 10.1186/s41232-022-00202-0 PMC9059385 35501926

[77] Domcke S , Shendure J . A reference cell tree will serve science better than A reference cell atlas. Cell 2023;186:1103–14. 10.1016/j.cell.2023.02.016 36931241

[78] Jeon OH , Kim C , Laberge R-M , et al . Local clearance of senescent cells attenuates the development of post-traumatic osteoarthritis and creates a pro-regenerative environment. Nat Med 2017;23:775–81. 10.1038/nm.4324 28436958 PMC5785239

[79] Jiang Y , Tuan RS . Origin and function of cartilage stem/progenitor cells in osteoarthritis. Nat Rev Rheumatol 2015;11:206–12. 10.1038/nrrheum.2014.200 25536487 PMC5413931

[80] Wang X , Ning Y , Zhang P , et al . Comparison of the major cell populations among osteoarthritis, Kashin–Beck disease and healthy chondrocytes by single-cell RNA-seq analysis. Cell Death Dis 2021;12:551. 10.1038/s41419-021-03832-3 34045450 PMC8160352

[81] McGinnis CS , Murrow LM , Gartner ZJ . DoubletFinder: Doublet Detection in Single-Cell RNA Sequencing Data Using Artificial Nearest Neighbors. Cell Syst 2019;8:329–37. 10.1016/j.cels.2019.03.003 30954475 PMC6853612

[82] Kim D , Paggi JM , Park C , et al . Graph-based genome alignment and genotyping with HISAT2 and HISAT-genotype. Nat Biotechnol 2019;37:907–15. 10.1038/s41587-019-0201-4 31375807 PMC7605509

[83] Liao Y , Smyth GK , Shi W . featureCounts: an efficient general purpose program for assigning sequence reads to genomic features. Bioinformatics 2014;30:923–30. 10.1093/bioinformatics/btt656 24227677

[84] Korsunsky I , Millard N , Fan J , et al . Fast, sensitive and accurate integration of single-cell data with Harmony. Nat Methods 2019;16:1289–96. 10.1038/s41592-019-0619-0 31740819 PMC6884693

[85] Welch JD , Kozareva V , Ferreira A , et al . Single-Cell Multi-omic Integration Compares and Contrasts Features of Brain Cell Identity. Cell 2019;177:1873–1887. 10.1016/j.cell.2019.05.006 31178122 PMC6716797

[86] Hao Y , Hao S , Andersen-Nissen E , et al . Integrated analysis of multimodal single-cell data. Cell 2021;184:3573–3587. 10.1016/j.cell.2021.04.048 34062119 PMC8238499

[87] Tran HTN , Ang KS , Chevrier M , et al . A benchmark of batch-effect correction methods for single-cell RNA sequencing data. Genome Biol 2020;21:12. 10.1186/s13059-019-1850-9 31948481 PMC6964114

[88] Stuart T , Butler A , Hoffman P , et al . Comprehensive Integration of Single-Cell Data. Cell 2019;177:1888–902. 10.1016/j.cell.2019.05.031 31178118 PMC6687398

[89] Qiu X , Mao Q , Tang Y , et al . Reversed graph embedding resolves complex single-cell trajectories. Nat Methods 2017;14:979–82. 10.1038/nmeth.4402 28825705 PMC5764547

[90] Yu G , Wang L-G , Han Y , et al . clusterProfiler: an R Package for Comparing Biological Themes Among Gene Clusters. OMICS: A Journal of Integrative Biology 2012;16:284–7. 10.1089/omi.2011.0118 22455463 PMC3339379

[91] Soul J , Dunn SL , Hardingham TE , et al . PhenomeScape: a cytoscape app to identify differentially regulated sub-networks using known disease associations. Bioinformatics 2016;32:3847–9. 10.1093/bioinformatics/btw545 27559157 PMC5167065

[92] Kamburov A , Wierling C , Lehrach H , et al . ConsensusPathDB--a database for integrating human functional interaction networks. Nucleic Acids Res 2009;37(Database issue):D623–8. 10.1093/nar/gkn698 18940869 PMC2686562

[93] Genomes Project C , Auton A , Brooks LD , et al . A global reference for human genetic variation. Nature 2015;526:68–74. 26432245 10.1038/nature15393 PMC4750478

[94] Chou C-H , Jain V , Gibson J , et al . Synovial cell cross-talk with cartilage plays a major role in the pathogenesis of osteoarthritis. Sci Rep 2020;10:10868. 10.1038/s41598-020-67730-y 32616761 PMC7331607

