# Supplementary material for: Unveiling inflammatory and prehypertrophic cell populations as key contributors to knee cartilage degeneration in osteoarthritis using multi-omics data integration
Source: Ann Rheum Dis. 2024 Feb 7;83(7):926–44. doi: 10.1136/ard-2023-224420 (PMC11187367; doi:10.1136/ard-2023-224420)
Supplement: Supplementary data [file ard-2023-224420supp002.zip › SupplTables.pdf]

Unveiling inflammatory and prehypertrophic cell populations as key contributors to knee cartilage degeneration in osteoarthritis using multi-omics data integration

Yue Fan[1,2,3], Xuzhao Bian[1], Xiaogao Meng[4,5,6], Lei Li[1], Laiyi Fu[7], Yanan Zhang[1], Long Wang[1,8], Yan Zhang[1,9], Dalong Gao[10], Xiong Guo[2], Mikko J. Lammi[11], Guangdun Peng[4,6,12#], and Shiquan Sun[1,2,3,#]

Supplementary Tables

Supplementary Table 1. The clinical information of subjects used for scRNA-seq and Geo-seq profiling in this study. SOA2-1, SOA2-2, SOA3-1, SOA3-2, SOA4-1, SOA4-2 and SC1 were obtained from the same individual with OA2-1, OA2-2, OA3-1, OA3-2, OA4-1, OA4-2 and C1 while used for spatial Geo-seq analysis.

Trait	Sample ID	Age	Gender	Protocol
**Control**	C1	54	Male	10X Genomics
	SC1			Geo-seq
**Control**	C2	46	Male	10X Genomics
**Control**	C3	55	Female	10X Genomics
**OA**	OA1-1	58	Female	10X Genomics
	OA1-2			
**OA**	SOA1-1	64	Female	Geo-seq
	SOA1-2			
**OA**	OA2-1	51	Female	10X Genomics
	OA2-2			
	SOA2-1			Geo-seq
	SOA2-2			
**OA**	OA3-1	61	Male	10X Genomics
	OA3-2			
	SOA3-1			Geo-seq
	SOA3-2			
**OA**	OA4-1	73	Male	10X Genomics
	OA4-2			
	SOA4-1			Geo-seq
	SOA4-2			
**OA**	OA5-1	61	Female	10X Genomics
	OA5-2			
**OA**	OA6-1	70	Male	10X Genomics
	OA6-2			
**OA**	OA7-1	75	Female	10X Genomics
	OA7-2			
**OA**	OA8-1	66	Male	10X Genomics
	OA8-2			

Supplementary Table 2. The parameter settings of quality control for 19 scRNA-seq data sets.

Sample ID	Min #Features	Max #Features	Max #UMI	%MT genes	#Cells	#Cells after QC	#Mediate genes
OA1-1	200	5000	20000	15	13,900	11,731	1,162
OA1-2	200	5000	20000	15	11,052	8,994	1,278
OA2-1	200	5000	15000	20	5,579	5,263	1,894
OA2-2	200	8000	40000	25	10,615	10,457	1,415
OA3-1	200	4000	20000	5	5,746	5,004	2,218
OA3-2	200	3500	20000	5	5,931	5,245	2,474
OA4-1	200	3500	10000	10	10,402	9,807	1,333
OA4-2	200	5000	25000	10	12,633	12,006	1,372
OA5-1	200	5500	20000	25	7,310	6,458	2,013
OA5-2	200	7500	50000	25	5,545	5,063	2,777
OA6-1	200	5000	30000	20	6,321	6,003	1,795
OA6-2	200	6000	30000	20	9,035	8,519	1,880
OA7-1	200	5000	30000	20	6,256	5,935	2,669
OA7-2	200	6000	30000	10	5,369	4,690	2,185
OA8-1	200	6000	30000	5	7,295	6,577	2198
OA8-2	200	5000	25000	5	8,771	7,637	2,051
C1	200	6000	50000	20	1,573	1,345	3,080
C2	100	5000	30000	20	10,305	8,183	2,151
C3	200	8000	40000	25	7,586	6,979	2,454

Supplementary Table 5. The summary of spatially resolved transcriptomic data profiled by Geo-seq.

Condition	Zone	#Spots	#Expressed genes	Mean Read depth
WB	AS	12	23,478	828,996
	SZ	17	21,654	397,837
	MZ	15	23,652	269,518
	DZ	9	22,831	453,665
NWB	AS	11	25,159	494,040
	SZ	14	23,788	331,085
	MZ	13	24,193	491,952
	DZ	12	24,665	572,465
Control	AS	7	18,432	279,282
	SZ	5	12,529	199,767
	MZ	4	14,113	262,630
	DZ	5	18,882	345,316

Supplementary Table 15. The clinical information of subjects used for Immunohistochemistry staining.

Trait	Sample ID	Age	Gender
OA	OA9	60	Female
OA	OA10	64	Male
OA	OA11	69	Female
OA	OA12	72	Male
OA	OA13	65	Female
OA	OA14	71	Male
Control	C4	37	Male
Control	C5	56	Female
Control	C6	50	Male
Control	C7	23	Male
Control	C8	39	Female
Control	C9	43	Female

Supplementary Table 16. Primers used in this study for Real-time analysis.

Gene	Forward primer (5'→3')	Reverse primer (5'→3')
MIF	CTGCACAGCATCGGCAAGAT	AGTTGATGTAGACCCTGTCCG
CD74	CAGTGCTATGGGAGCATCGG	GCTCTCACATGGGGACTGGG
IL-1B	AACCTCTTCGAGGCACAAGG	GTCCTGGAAGGAGCACTTCAT
TNF-α	TCTCCTTCCTGATCGTGGCA	CAGCTTGAGGGTTTGCTACAAC
β-actin	ACAGAGCCTCGCCTTTGC	CCACCATCACGCCCTGG
