# Supplementary material for: Unveiling inflammatory and prehypertrophic cell populations as key contributors to knee cartilage degeneration in osteoarthritis using multi-omics data integration
Source: Ann Rheum Dis. 2024 Feb 7;83(7):926–44. doi: 10.1136/ard-2023-224420 (PMC11187367; doi:10.1136/ard-2023-224420)
Supplement: Supplementary data [file ard-2023-224420supp001.pdf]

Unveiling inflammatory and prehypertrophic cell populations as key contributors to knee cartilage degeneration in osteoarthritis using multi-omics data integration

Yue Fan[1,2,12], Xuzhao Bian[1,2,12], Xiaogao Meng[4,5,6,12], Lei Li[1,2], Laiyi Fu[7,8], Yanan Zhang[1,2], Long Wang[1,2], Yan Zhang[1,2,9], Dalong Gao[10], Xiong Guo[1,2], Mikko J. Lammi[11], Guangdun Peng[4,6,#], and Shiquan Sun[1,2,3,#]

Supplementary Figures

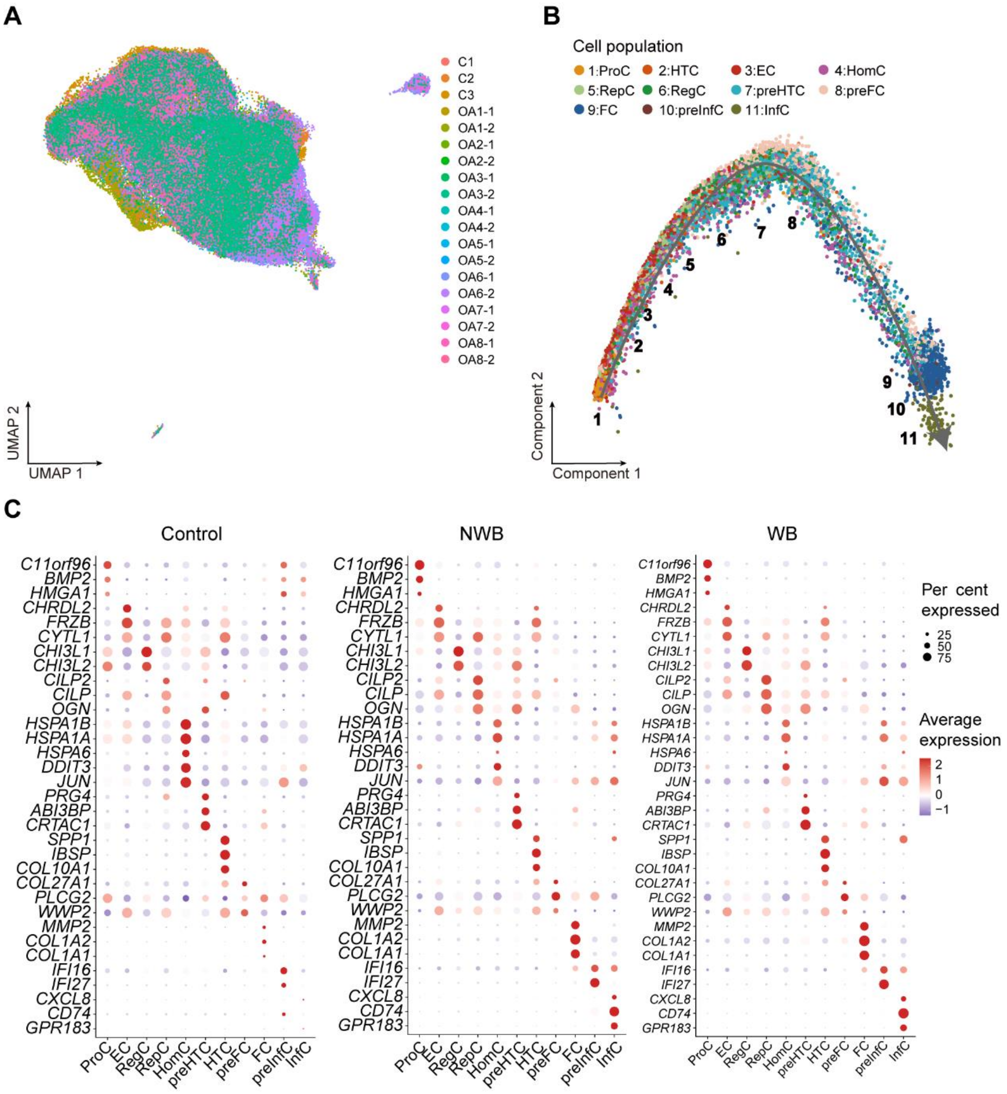

Supplementary Figure 1. The single cell landscape of human knee cartilage in patients with WB, NWB, and non-OA controls. (**A**) The visualization of a total of 135,896 chondrocytes from all samples. The chondrocytes were colored according to the sample involved. (**B**) The trajectory inference analysis to show the chondrocytes differentiation path, i.e., ProCs (yellow) are at the start position, the FC (blue), preInfC(crimson), and InfC population (brown) are located at the termination of the chondrocyte pseudotime inferred by Monocle 2. (**C**) The dot plot shows the mean expressions of the marker genes we defined for 11 major cell populations in the control(left panel), NWB (middle panel), and WB (right panel) samples. The color represents the average scaled gene expression level (z-score) and the dot size represents the percentage of cells that the marker gene detected in each cell population.

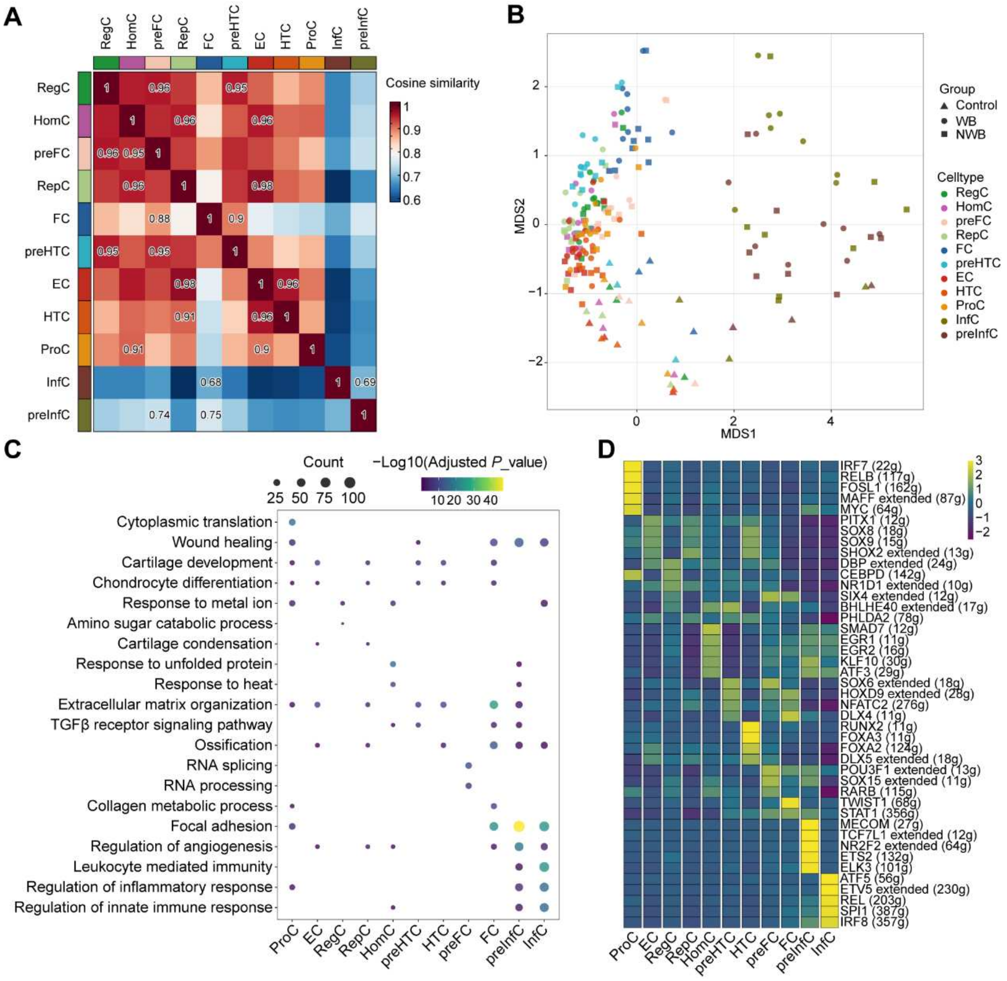

Supplementary Figure 2. The complementary results of Figure 1. (**A**) Heatmap showed the transcriptional similarity among 11 chondrocyte populations. The transcriptional signatures are highly correlated among 8 common populations. (**B**) Pseudobulk-level multidimensional scaling (MDS) plot. Each point represents one chondrocyte population-sample instance. Points are colored by chondrocyte population and shaped by groups (WB, NWB, and control). Different chondrocyte populations from the same group get closer on the MDS plot. (**C**) Dot plot showed the significant GO terms enriched by the up-regulated genes in each chondrocyte population, the color scale is the adjusted *P-value* and the size scale is the number of genes enriched in each GO term. (**D**) Heatmap shows the TF regulons per chondrocyte population generated from the SCENIC analysis.

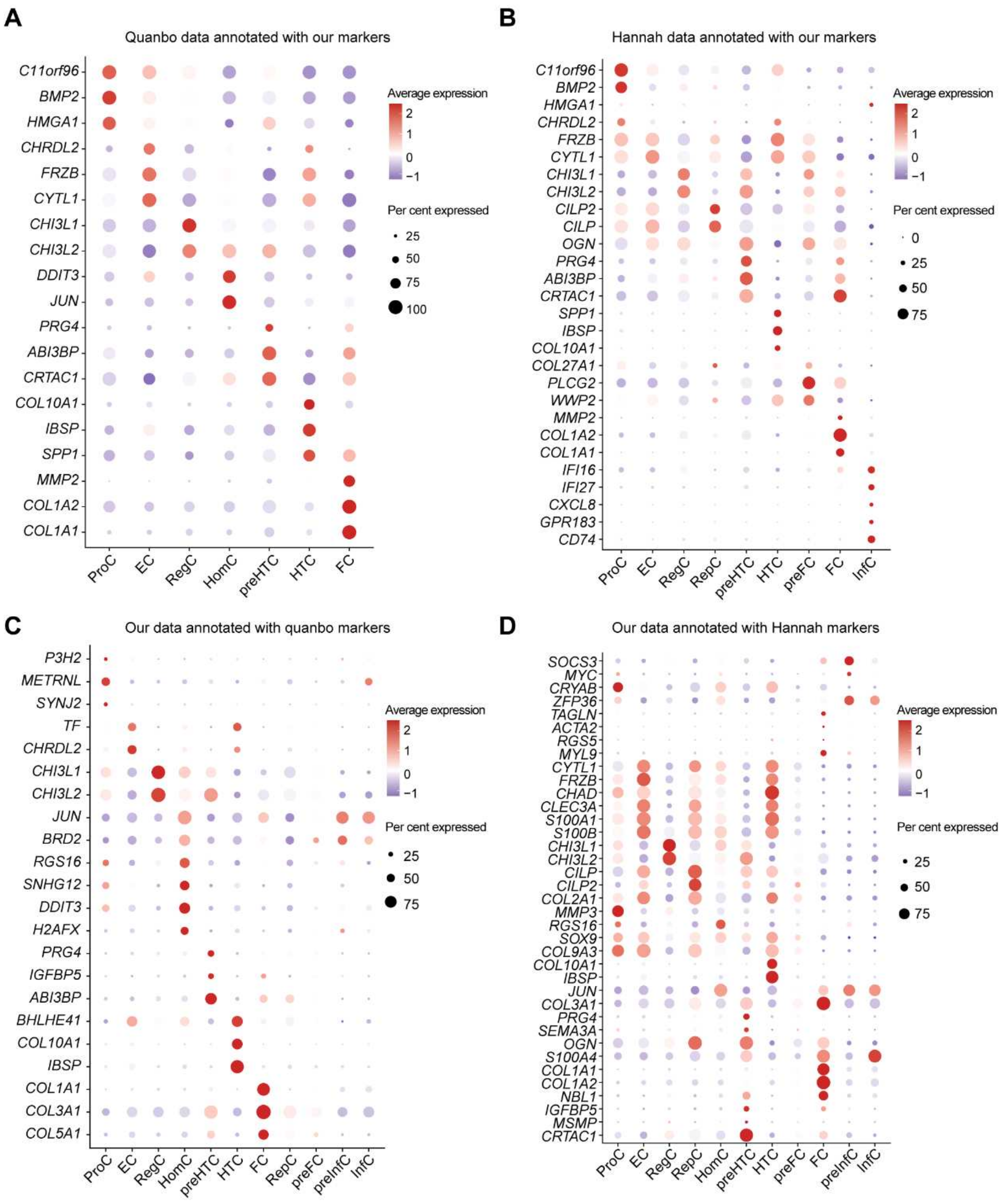

Supplementary Figure 3. The identified marker genes were obtained from our single-cell landscape mapping in the GSE104782 and GSE220243 external datasets. (A-B) The dot plot shows the annotation and mean expressions of the marker genes we defined in the **(A)** GSE104782 and **(B)** GSE220243 external scRNA-seq dataset of Osteoarthritis knee cartilage. **(C-D)** The dot plot shows the annotation and mean expressions of the marker genes defined by **(C)** GSE104782 and **(D)** GSE220243 external dataset of Osteoarthritis knee cartilage in our scRNA-seq data.

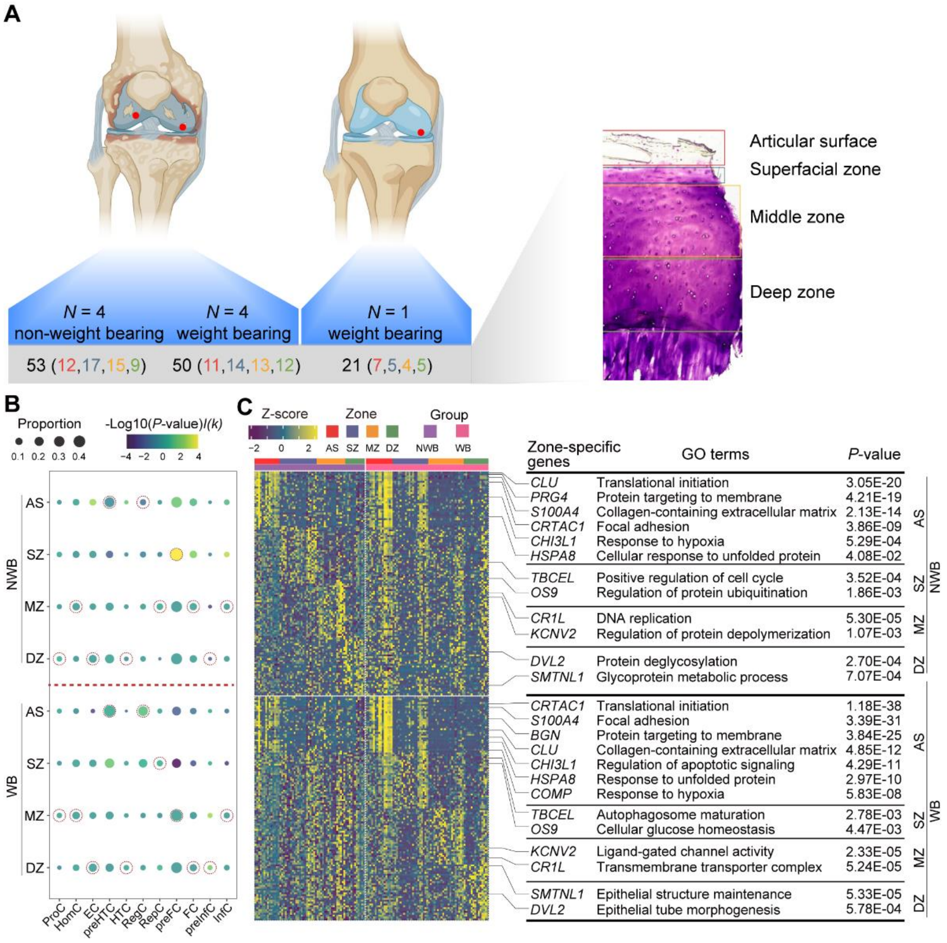

Supplementary Figure 4. The spatial transcriptomic landscape of human knee cartilage from WB and NWB. (**A**) Schematic representation of zone-specific cartilage collection for Geo-seq. The articular cartilage was classified into 4 different zones, namely, articular surface (AS), superficial zone (SZ), middle zone (MZ), and deep zone (DZ). The zone classification was based on chondrocyte morphology. To avoid overlap between zones, approximately 200μm regions between different zones were discarded. **(B)** Cell compositions for each zone mapped spatial landscape by CIBERSORTx. The dot plot showed the cell composition difference between WB and NWB samples. Gene signature matrices were constructed based on scRNA-seq data. The dot size represents the proportion of each chondrocyte population in a particular zone and the color represents the -log10(*P*-value) (permutation *t*-test). *I(k)* represents an indicator function, where *I(k) = 1* if a chondrocyte population is abundant in the WB and *I(k) = -1* if a chondrocyte population is abundant in the NWB sample. The dot which represents the highest cell proportion for each chondrocyte population across 4 zones is highlighted by dotted circles. (**C**) The heatmap displays the expression levels of the top 10 zone-specific genes in AS (red), SZ (purple), MZ (orange), and DZ (green) for NWB (upper panel) or WB samples (bottom panel). The GO terms were enriched by the zone-specific genes of NWB (upper panel) or WB (bottom panel) samples. Zone-

specific genes of MZ and DZ were not enriched in any OA-related meaningful pathways. LCM: laser capture microdissection.

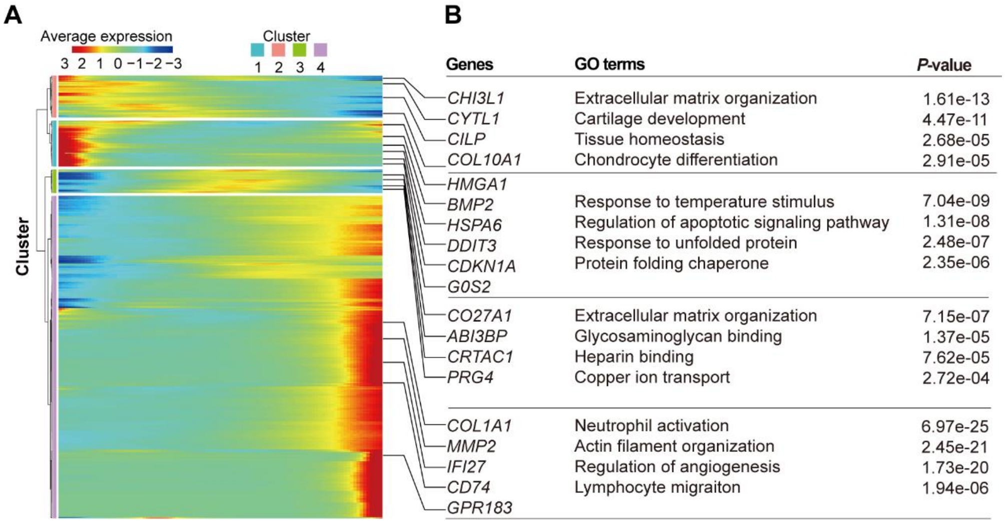

Supplementary Figure 5. Pseudotime analysis shows inflammatory signature was mainly present in the termination of the differentiation process (A) The heatmap shows the relative expression patterns of the top marker genes along the inferred pseudotime, which can be grouped into four clusters according to the gene expression levels on pseudotime. (**B**) The significant GO terms were enriched by the pseudotime DE genes of four clusters.

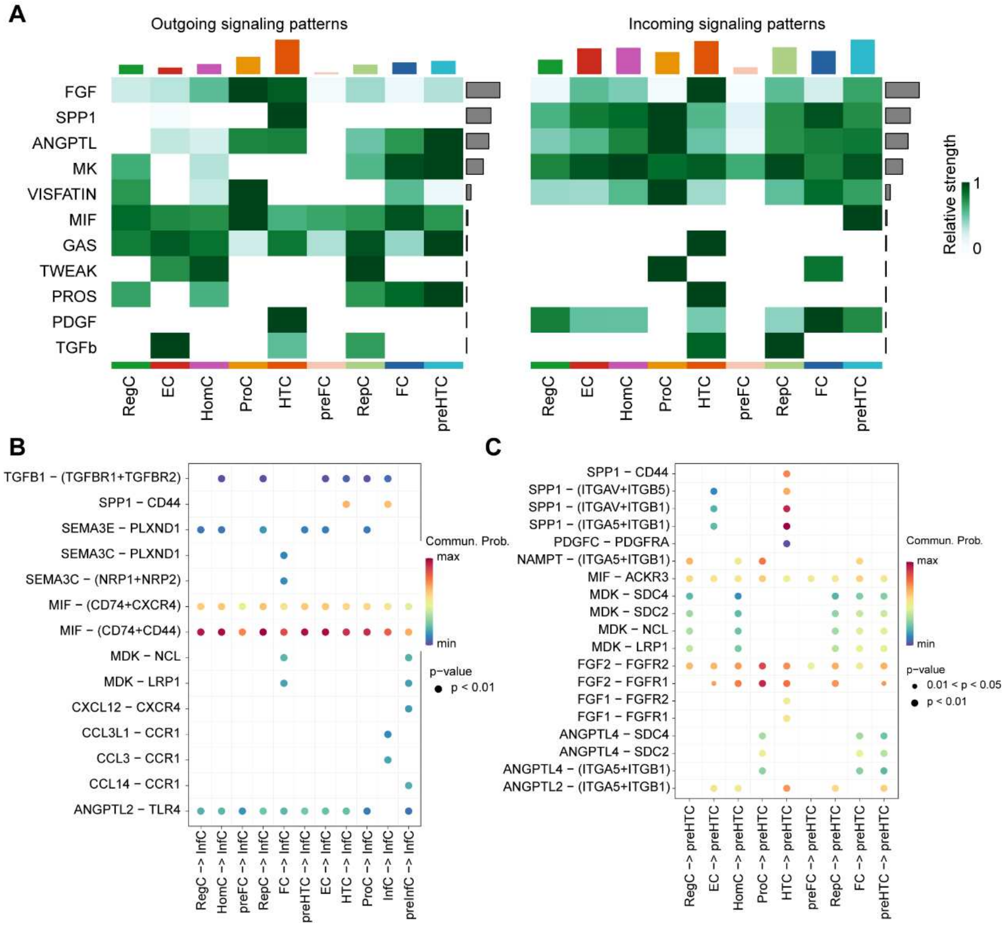

Supplementary Figure 6. The significant ligand-receptor pairs that contribute to the MIF pathways. (**A**) A heatmap shows cell-cell communications in non-OA control samples. The grey bar on the y-axis denotes the relative strength of the predicted signaling pathway in the overall network while the color bar on the x-axis represents the relative contribution of the chondrocyte population to the overall signaling milieu (top x-axis). (**B**) The bubble plot shows the significant interactions (L-R pairs) among InfC and other chondrocyte populations inferred by CellChat for OA samples. InfC interacted with other chondrocyte populations mainly through MIF-CD74 ligand-receptor pairs. (**C**) The bubble plot shows the significant interactions (L-R pairs) among preHTC and other chondrocyte populations inferred by CellChat for Control samples. preHTC interacted with other chondrocyte populations through MIF-ACKR3 ligand-receptor pairs instead of MIF-CD74 ligand-receptor pairs.

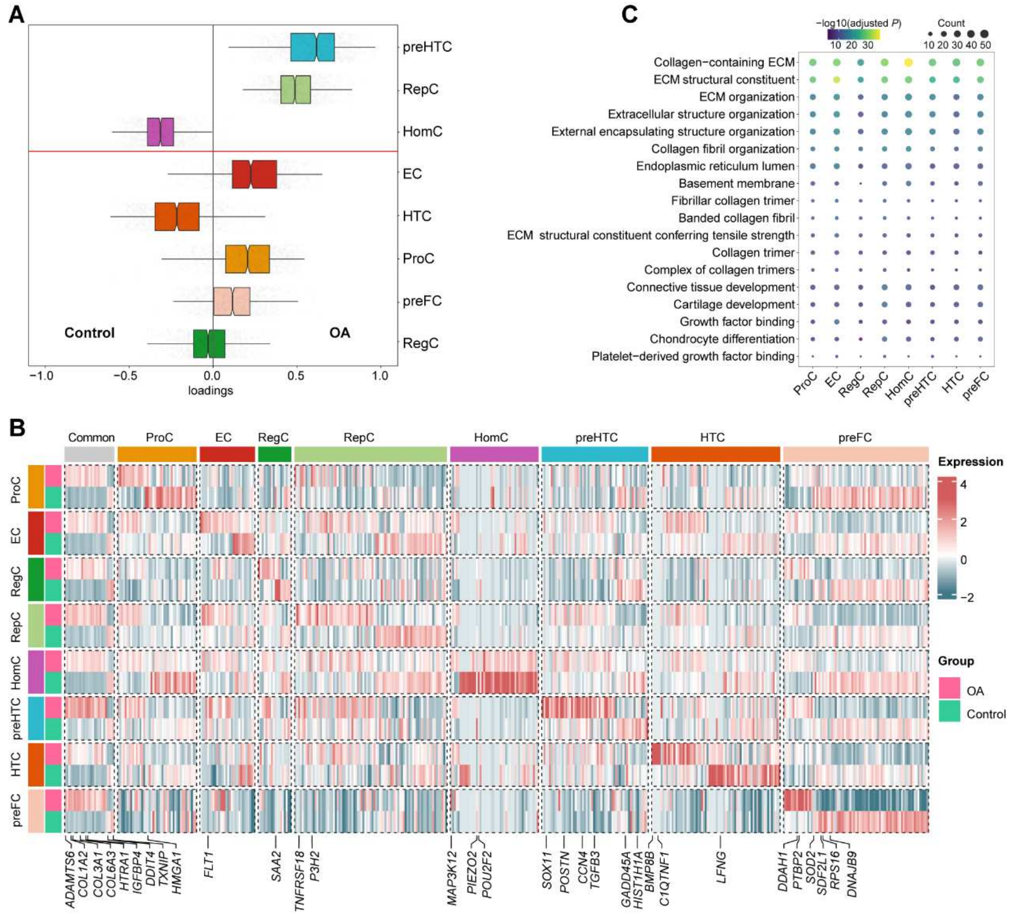

Supplementary Figure 7. **The complementary results of Figure 4.** (**A**) CoDA (compositional data analysis) loadings (x-axis) of the cell types (y-axis) in the scRNA-seq data obtained from Cocao. Positive loadings correspond to over-representation in the OA samples and negative loadings mean over-representation in Control. The red horizontal line separates cell types passing the significance threshold (adjust *P* < 0.05 after BH correction). (**B**) Heatmap of pseudobulk gene expression profiles of DE genes detected in a single chondrocyte population or across all chondrocyte populations. For each gene, colored column bars indicate chondrocyte populations in which DE genes were differentially expressed. Colored row bars indicate chondrocyte populations and OA-control status. (**C**) The significant GO terms were enriched by the up-regulated genes in each chondrocyte population between OA and control samples, the color scale is the adjusted *P-value* and the size scale is the number of genes enriched in each GO term. Deregulated ECM metabolism, cell proliferation, and differentiation were the common transcriptome landscape of OA chondrocytes.

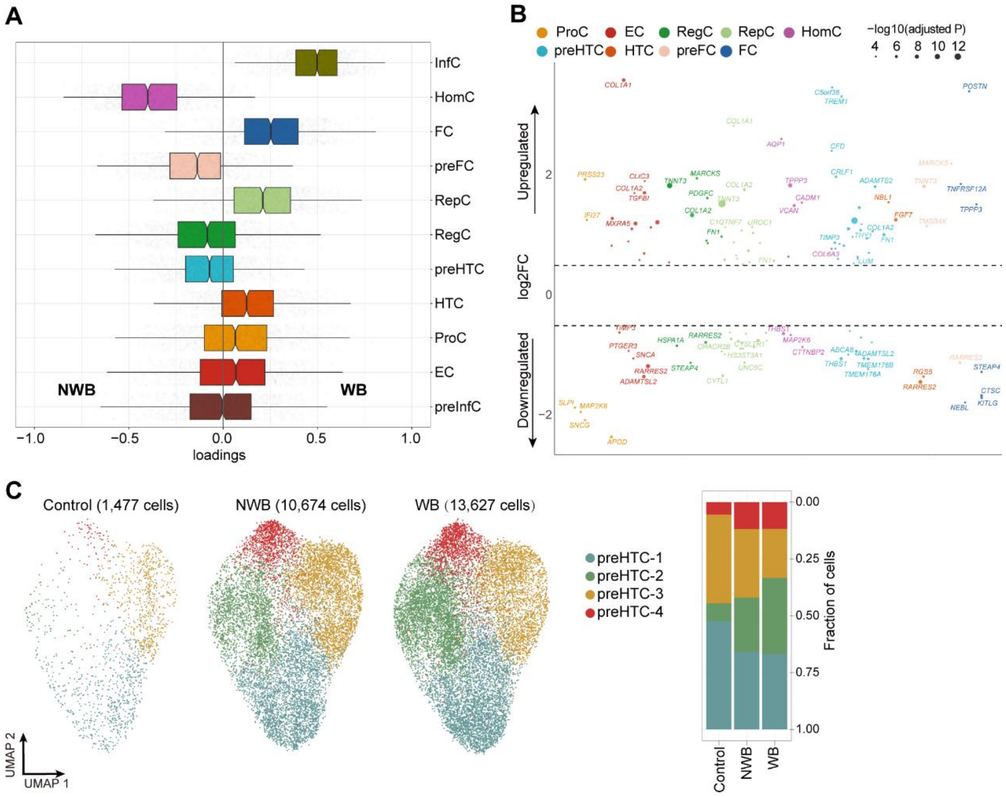

Supplementary Figure 8. **The cellular composition and expression shifts between WB and NWB.** (**A**) CoDA (compositional data analysis) loadings (x-axis) of the cell types (y-axis) in the scRNA-seq data obtained from Cocao. Positive loadings correspond to over-representation in the WB and negative loadings mean over-representation in NWB. No chondrocyte population passed the significance threshold (adjusted P < 0.05 after BH correction). (**B**) The scatter plot demonstrates the population-specific DE genes (including up-regulated and down-regulated) between WB and NWB samples. The dot size represents the value of -log10 (adjusted *P*). (**C**) The visualization of chondrocyte populations using UMAP projections, where a total of 25,778 preHTCs were split by groups. There are a total of 4 subpopulations, namely preHTC-1 (blue), preHTC-2 (green), preHTC-3 (yellow) and preHTC-4 (red). The bar plot shows the corresponding subpopulation compositions among the WB, NWB, and non-OA controls for each cell population.

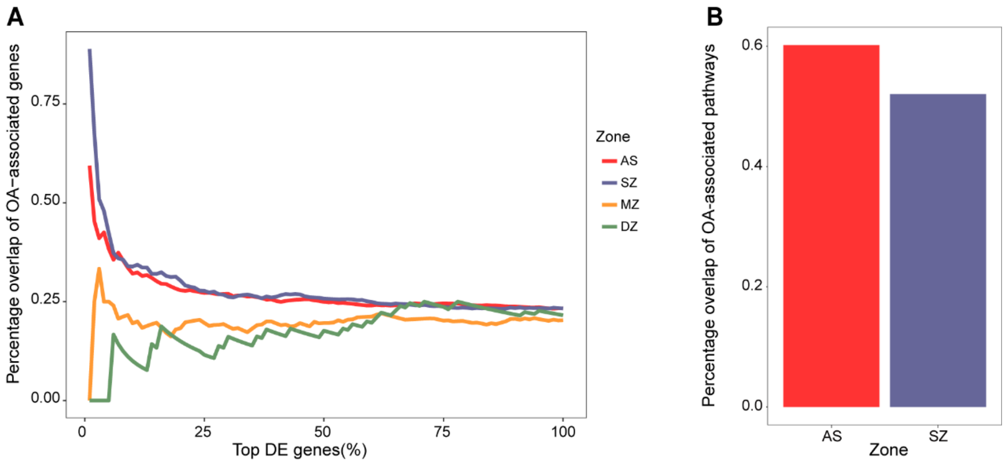

Supplementary Figure 9. **The validation of zone-specific DE genes between patients with OA and non-OA controls.** (**A**) The percentage overlap of detected OA up-regulated zone-specific DE genes with OA-associated genes. The OA-associated genes were obtained through bibliographic search from PubMed. The top ranked AS- and SZ-specific DE genes were more enriched in OA-associated genes. (**B**) The percentage overlap of pathways enriched by OA up-regulated zone-specific DE genes with OA-associated pathways. The OA-associated pathways were obtained through bibliographic search from PubMed. Most of the pathways were validated by previous studies. DE genes from MZ and DZ were not enriched in any GO terms and therefore been excluded.

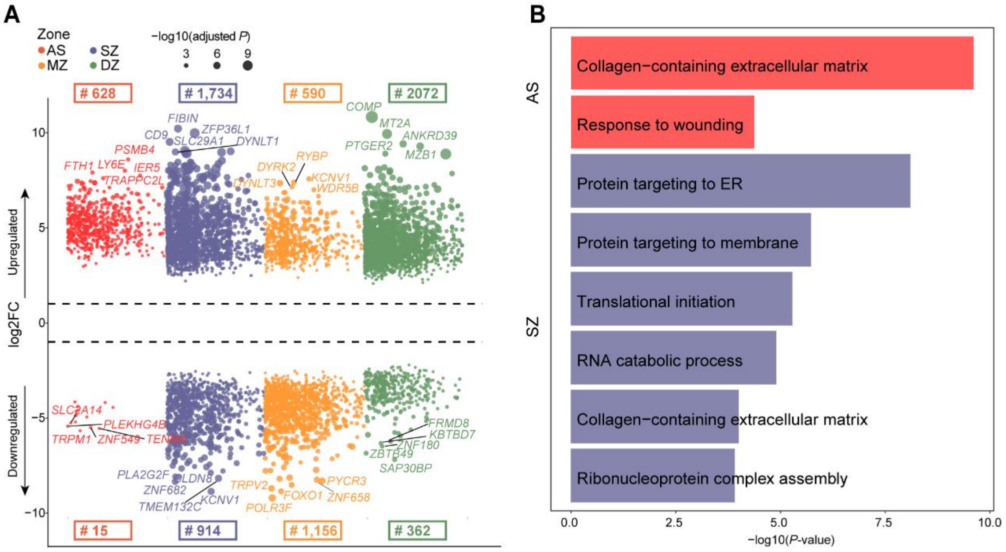

Supplementary Figure 10. The differential expression and enrichment analyses of each zone of Geo-seq data between WB and NWB. (**A**) The scatter plot shows the zone-specific DE genes between patients with WB and NWB samples, i.e., articular surface (red), superficial zone (yellow), middle zone (blue), and deep zone (green). The numbers on the top panel represent the number of significant DE genes for each zone. (**B**) The bar plot shows the significant GO terms that were enriched by the zone-specific DE genes. Notably, the MZ- and DZ-specific DE genes were not enriched in any biologically meaningful pathways.

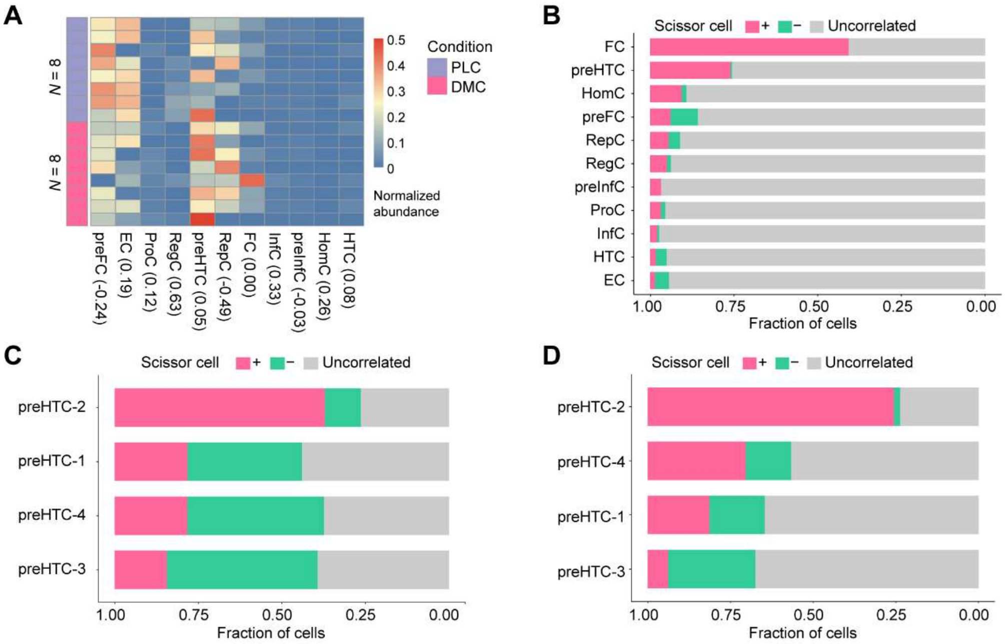

Supplementary Figure 11. The results were obtained from integration with scRNA-seq and bulk RNA-seq data. (**A**) The heatmap shows cell compositions of 8 PLC (un-weighted-bearing) and paired DMC (weighted-bearing) from E-MTAB-4304. Spearman correlation suggests that preFCs are negatively correlated with WB status. (**B**) Bar plot depicting the fraction of Scissor+(positively correlated), Scissor-(negatively correlated), and uncorrelated cells with OA with the 11 chondrocyte populations of articular cartilage in the E-MTAB-4304 dataset. The red/green bar represents the proportion of each chondrocyte population that significantly positively/negatively correlated with WB status and the gray bar represents the proportion of each chondrocyte population that is not correlated with WB status. (**C-D**) The bar plot demonstrates the fraction of Scissor+ (positively correlated), Scissor- (negatively correlated), and uncorrelated cells with OA with the 4 preHTC subpopulations of articular cartilage in the (**C**) GSE114007 data set and (**D**) E-MTAB-4304 data set.

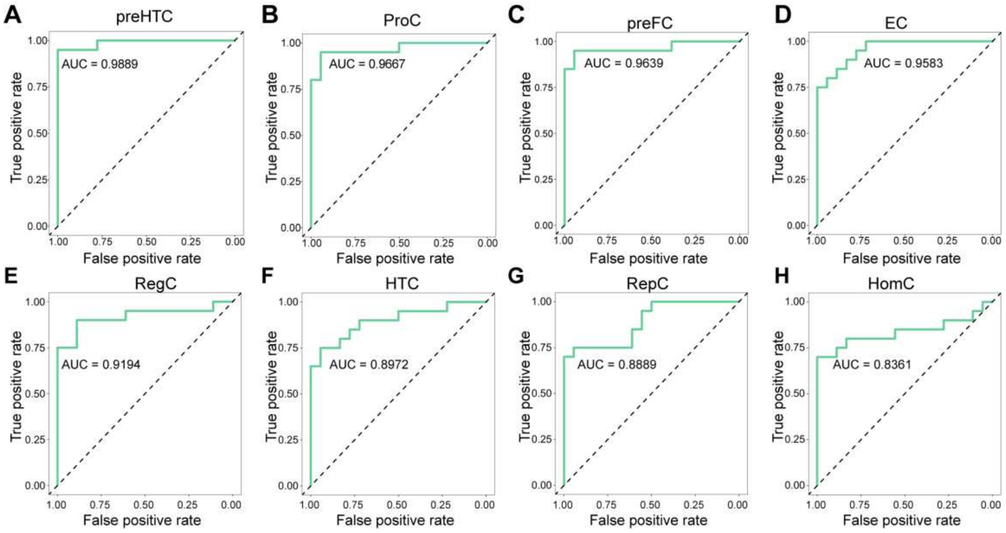

Supplementary Figure 12. The receiver operating curves (ROC) represent the ability of population-specific DE genes to predict OA status. The classification model was trained by logistic regression models with pre-selected genes from (**A**) preHTC-specific DE genes, AUC=0.9889 (**B**) ProC-specific DE genes, AUC=0.9667. (**C**) preFC-specific DE genes, AUC = 0.9639. (**D**) EC-specific DE genes, AUC = 0.9583. (**E**) RegC-specific DE genes, AUC = 0.9194. (**F**) HTC-specific DE genes, AUC = 0.8972. (**G**) RepC-specific DE genes, AUC = 0.8889. (**H**) HomC-specific DE genes, AUC = 0.8361.

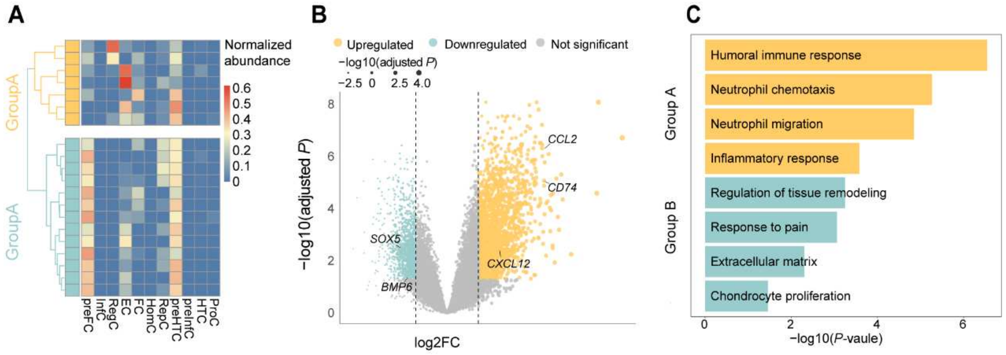

Supplementary Figure 13. The stratification of OA patients for bulk RNA-seq data (GSE114007). (**A**) The heatmap illustrates the two subtypes of 20 OA samples identified by hierarchical cluster with ward.D2 parameter. 20 samples are clustered into 2 subtypes, an inflammation-related subtype with more fraction of FCs and a noninflammatory-related subtype with more fraction of preFCs (**B**) The volcano plot shows the differentially expressed genes between two subtypes of OA, i.e., Group A versus Group B. (**C**) The significant GO terms were enriched by the top-up/down-regulated DE genes. The upregulated genes in Group A (inflammation-related subtype) were mainly involved in inflammatory and immune response-associated pathways, while Group B (noninflammatory-related subtype) up-regulated genes were mainly involved in chondrocyte proliferation pathways. This result further validates the OA patient stratification for 131 OA patients.

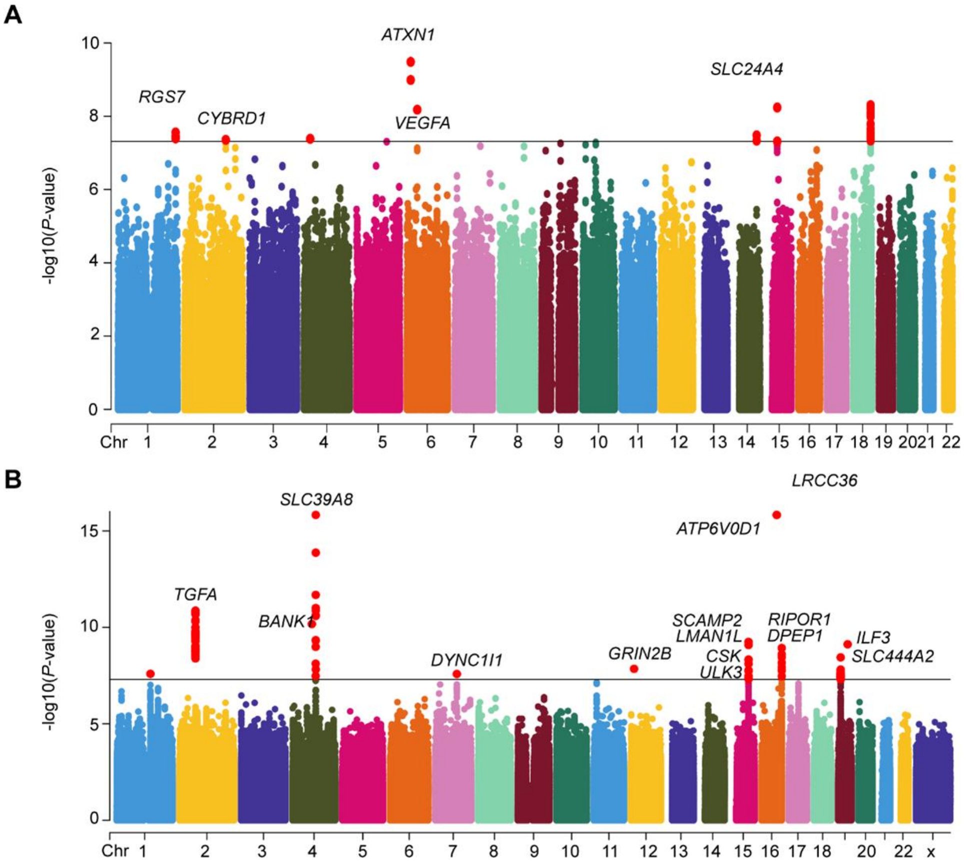

Supplementary Figure 14. The Manhattan plot shows the GWAS results for knee pain from Asian and European ancestry from UK Biobank data. (**A**) The Manhattan plot shows the GWAS associations of OA pain that are only from the East Asian ancestry of the UK Biobank. The summaries were obtained by performing the GMMAT method. (**B**) The GWAS summary data were obtained from Neale's Laboratory and performed by linear regression models. The y-axis represents the negative logarithm (base 10) of the variant *P*-value and the x-axis represents the position on the chromosome.

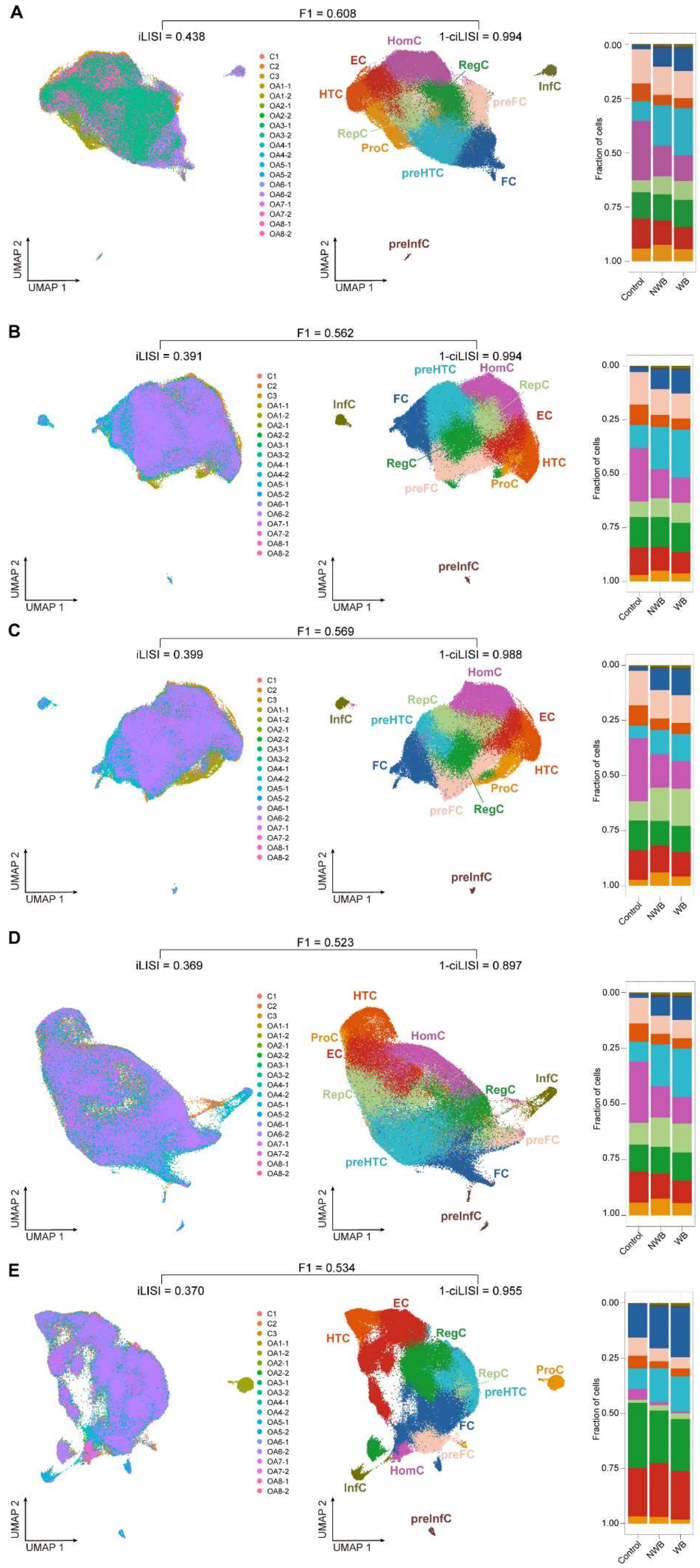
A
F1 = 0.608
iLISI = 0.438
1-ciLISI = 0.994
C1
C2
C3
OA1-1
OA1-2
OA2-1
OA2-2
OA3-1
OA3-2
OA4-1
OA4-2
OA5-1
OA5-2
OA6-1
OA6-2
OA7-1
OA7-2
OA8-1
OA8-2
HomC
EC
RegC
HTC
InfC
preFC
RepC
ProC
preHTC
FC
preInfC
UMAP 2
UMAP 1
Fraction of cells
0.00
0.25
0.50
0.75
1.00
Control
NWB
WB
B
F1 = 0.562
iLISI = 0.391
1-ciLISI = 0.994
C
F1 = 0.569
iLISI = 0.399
1-ciLISI = 0.988
D
F1 = 0.523
iLISI = 0.369
1-ciLISI = 0.897
E
F1 = 0.534
iLISI = 0.370
1-ciLISI = 0.955

Supplementary Figure 15. Quantitative evaluation of the batch effects correction across different parameters and methods via LISI metric. (**A-E**)The visualization of chondrocyte split by samples in the first column, the visualization of chondrocyte split by clusters in the second column, and the bar plots show the corresponding chondrocyte compositions for Control, NWB, and WB. (**A-C**) The integrative analysis was performed using Harmony, the parameter settings are (**A**) PCs=30, resolution=0.9 , (**B**) PCs=20, resolution=0.9 or (**B**) PCs=40, resolution=0.9; (**D**) The integrative analysis was performed using Seurat, the parameter settings are PCs = 30, resolution=0.8. (**E**) The integrative analysis was performed using LIGER, the parameter settings are PCs = 30, and resolution = 0.7. iLISI, 1-cLISI, and the combined F1 score are annotated as plot titles. A higher F1 score indicates better batch effects correction performance. The F1 score of the above integration parameters or methods was inferior to our integration results (**A**), where the F1 score = 0.608.
